# Characterization of *FBA* genes in potato (*Solanum tuberosum* L.) and expression patterns in response to light spectrum and abiotic stress

**DOI:** 10.3389/fgene.2024.1364944

**Published:** 2024-04-12

**Authors:** Ting Li, Xinyue Hou, Zhanglun Sun, Bin Ma, Xingxing Wu, Tingting Feng, Hao Ai, Xianzhong Huang, Ruining Li

**Affiliations:** ^1^ Center for Crop Biotechnology, College of Agriculture, Anhui Science and Technology University, Fengyang, China; ^2^ Country College of Life Sciences, Shihezi University, Shihezi, China

**Keywords:** fructose-1, 6-bisphosphate aldolase, genome-wide identification, light spectrum, abiotic stress, gene expression, *Solanum tuberosum*

## Abstract

Fructose-1, 6-bisphosphate aldolase (FBA) plays vital roles in plant growth, development, and response to abiotic stress. However, genome-wide identification and structural characterization of the potato (*Solanum tuberosum* L.) FBA gene family has not been systematically analyzed. In this study, we identified nine *StFBA* gene members in potato, with six *StFBA* genes localized in the chloroplast and three in the cytoplasm. The analysis of gene structures, protein structures, and phylogenetic relationships indicated that *StFBA* genes were divided into Class I and II, which exhibited significant differences in structure and function. Synteny analysis revealed that segmental duplication events promoted the expansion of the *StFBA* gene family. Promoter analysis showed that most *StFBA* genes contained *cis*-regulatory elements associated with light and stress responses. Expression analysis showed that *StFBA3*, *StFBA8,* and *StFBA9* showing significantly higher expression levels in leaf, stolon, and tuber under blue light, indicating that these genes may improve photosynthesis and play an important function in regulating the induction and expansion of microtubers. Expression levels of the *StFBA* genes were influenced by drought and salt stress, indicating that they played important roles in abiotic stress. This work offers a theoretical foundation for in-depth understanding of the evolution and function of *StFBA* genes, as well as providing the basis for the genetic improvement of potatoes.

## 1 Introduction

Plants utilize various enzymes within their bodies to absorb solar energy and convert CO_2_ into organic carbon in nature (Wildman, 2002). Ribulose-1, 5-bisphosphate carboxylase/oxygenase (Rubisco) plays a crucial role in carbon fixation and regulates biomass synthesis during photosynthesis (Ashida et al., 2019). However, the low catalytic efficiency of Rubisco remains a major limiting factor in photosynthesis (Bracher et al., 2017). With the rise in global temperatures and CO_2_ concentrations, Rubisco is susceptible to oxidation, resulting in substantial loss of photosynthetic products. Some plants conserve energy by reducing the synthesis of Rubisco proteins when grown in high CO_2_ environments (Jordan and Ogren, 1984; Long et al., 2004). Previous studies have shown that increasing the levels of enzymes associated with the regeneration phase of ribulose-1, 5-bisphosphate (RuBP) in the Calvin cycle, such as sedoheptulose-1, 7-bisphosphatase (SBP), transketolase, and aldolase, can enhance plant carbon assimilation efficiency (Raines, 2003; Lefebvre et al., 2005). Among them, aldolase reaches its highest activity level under CO_2_ saturation (Haake et al., 1999). Overexpression of a gene-encoding plastid aldolase in transgenic tobacco enhanced the activity of the enzyme, accelerated the regeneration of RuBP, and the biomass significantly increased with increasing CO_2_ levels (Uematsu et al., 2012).

Fructose-1, 6-bisphosphate aldolase (FBA; EC 4.1.2.13) is an important enzyme involved in plant photosynthesis and energy metabolism (Carrera et al., 2021). It catalyzes the condensation of glyceraldehyde-3-phosphate (GAP) and dihydroxyacetone phosphate (DHAP) to fructose-1, 6-bisphosphate (FBP) during glycolysis and its reversible reaction, which is ultimately used for the regeneration of RuBP (Zgiby et al., 2000; Capodagli et al., 2014). FBA can be divided into Class I and Class II. Class I aldolase is primarily found in eukaryotes, while Class II aldolase is widely distributed in bacteria, saccharomycetes, and fungi. There are significant differences in the structure and function of these classes (Marsh and Lebherz, 1992; Izard and Sygusch, 2004; Galkin et al., 2007). In higher plants, Class I aldolase is composed of homotetramers (Lebherz et al., 1984; Perham, 1990) and its protein tertiary structure contains a typical conserved structural domain, called the TIM barrel (Hester et al., 1991; Blom and Sygusch, 1997). The TIM barrel is a conserved protein fold pattern, which consists of eight repetitive α-helices and eight β-folds, which are arranged alternately, forming a characteristic α/β-barrel structure present in the protein’s main peptide segment (Lorentzen et al., 2003; Frenkel and Trifonov, 2005). In addition, there are two subtypes of Class I aldolase: chloroplast aldolase, and cytoplasmic aldolase (Pelzer-Reith et al., 1993; Tsutsumi et al., 1994). Chloroplast aldolase is a bifunctional enzyme in the Calvin cycle, involved in the synthesis of FBP and sedoheptulose-1, 7-bisphosphate (SuBP). It catalyzes the generation of metabolic products in the process of starch synthesis (Sonnewald et al., 1994; Flechner et al., 1999). Furthermore, cytoplasmic aldolase is an important metabolic enzyme in the pathways of glycolysis and gluconeogenesis, where it catalyzes the production of FBP and participates in the synthesis of sucrose (Gross et al., 1999; Fan et al., 2009). Mutations in *Arabidopsis* cytoplasmic aldolase *AtFBA3* have been reported to affect the plastid glycolytic metabolism in the roots of plants, thereby limiting the synthesis of essential compounds such as amino acids (Carrera et al., 2021).

Previous studies reported that a slight reduction in FBA activity significantly inhibited the photosynthetic and carbon assimilations of antisense transgenic plants, which affected the synthesis of starch and severely inhibited the growth of plants (Jacquot et al., 1995; Haake et al., 1998; Bryant, 2000; Henkes et al., 2001), while reduced activity of *FBA* in tomato plants led to smaller seed size and slower plant growth (Cai et al., 2018). Suppressing the expression of the chloroplast *FBA* gene affects the development of tomatoes (Obiadalla-Ali et al., 2004). In rice, the *OsALD*-*Y* (fructose-1, 6-bisphosphate aldolase) encodes an FBA protein, which affects photosynthesis and sugar metabolism (Zhang et al., 2016). Furthermore, the *FBA* gene is also involved in a variety of physiological and biochemical processes, such as the regulation of secondary metabolism (Zeng et al., 2014), and regulation of plant growth and development (Haake et al., 1998; Obiadalla-Ali et al., 2004; Mutuku and Nose, 2012), responding to various biotic and abiotic stress (Lu et al., 2012; Cai et al., 2018), to regulate plant responses to hormones such as abscisic acid (Osakabe et al., 2005) and gibberellin (Konishi et al., 2004), as well as responses to environmental light signals (Oelze et al., 2014). It is therefore possible that *FBA* genes play a crucial role in enhancing the photosynthetic efficiency of plants, to improve crop yield and quality.

Potato is one of the four largest food crops worldwide (Zhang et al., 2017), and a significant crop for both food and as a vegetable in our country. The available genome sequences of potatoes (Pham et al., 2020; Zhou et al., 2020; Zhang et al., 2021) facilitate their use in potato growth and development. Members of the FBA gene family have been identified in different species, with eight identified in *Arabidopsis thaliana* (Lu et al., 2012), eight in *Solanum lycopersicum* (Cai et al., 2016), 22 in *Brassica napu*s (Zhao et al., 2019), 16 in *Nicotiana tabacum* (Zhao et al., 2021), and 17 in cotton (Li et al., 2021). When expression of potato chloroplast aldolase (Registry Number: Y10380) was inhibited using antisense repression of expression technology, the photosynthesis of plants was decreased to significantly affect the sugar and starch contents of potatoes (Haake et al., 1999). However, there is still a lack of systematic genome-wide identification and functional investigation of potato FBA gene family members. In this study, we identified nine *StFBA* genes using bioinformatics tools, to systematically analyze gene structures, protein structures, chromosome distributions, phylogenetic relationships, synteny, collinearities, and *cis*-acting elements in promoter regions. Furthermore, we determined the expression patterns of *StFBA* in different tissues and in response to abiotic stress. The results provide a theoretical basis for the in-depth use of *StFBA* functions to improve photosynthetic efficiency and tuber development in potatoes.

## 2 Materials and methods

### 2.1 Plant materials

Potato (cv. *Shepody*) was used as experimental material, provided by the Crop Biotechnology Research Center of Anhui University of Science and Technology. Under sterile conditions, potato plantlets were cut into 1–1.5 cm segments with leaves and then placed into medium in tissue culture bottles (inner diameter, 63 mm; height, 85 mm). Each bottle contained four stem segments and was placed in a tissue culture room with relative humidity of 65% ± 5%, daytime temperature of 22°C ± 2°C, night temperature of 18°C ± 2°C, photoperiod uniformly set to 8 h light/16 h dark, and photosynthetic photon flux density of 65 μmol m^−2^ s^−1^. The medium used for propagation of plantlets *in vitro* was solid Murashige and Skoog (MS) medium (4%, w/v, sucrose, 0.9%, w/v, agar). The medium used for induction of microtubers was solid, higher sucrose MS medium (8%, w/v, sucrose, 0.9%, w/v, agar). Potato plantlets grown for 30 days were acclimated and transplanted to pots containing vegetative soil and vermiculite (1:1) and placed in a plant growth room where the relative humidity was 65% ± 5%, the temperature was 22°C ± 2°C, and the photoperiod was uniformly set at 12 h light/12 h dark. Light intensity was 200 μmol m^−2^ s^−1^. The flower tissue, petal, stamen, and pistil at 45 days of growth, as well as the leaf, petiole, stem, stolon, root, and tuber at 60 days of growth were collected for tissue expression analysis of the *StFBA* gene.

### 2.2 Identification of *StFBA* members in potato

The whole genome sequence, protein and encode sequences of potato v6.1 were downloaded from the Phytozome v13 website (https://phytozome.jgi.doe.gov/pz/portal.html). The protein sequences of the Arabidopsis FBA gene family were sourced from the TAIR database (http://www.arabidopsis.org). Genomic data for rice, tomato, eggplant, tobacco, and wheat were acquired from the Rice Genome Annotation Project (RGAP, http://rice.plantbiology.msu.edu/) and Ensemble database (http://plants.ensembl.org/index.html), reserved for subsequent analysis. Algorithm based on BLASTP was performed using the amino acid sequences of *A*. *thaliana* FBA proteins as queries in the protein databases of *S*. *tuberosum*, with an *E*-value ≤ 1e-5 and other parameters as default values, and redundant sequences were manually deleted. The Hidden Markov Model (HMM) of Glycolytic (PF00274) and fructose-bisphosphate aldolase class II domain (PF01116) were downloaded from InterPro database (https://www.ebi.ac.uk/interpro/search/sequence/) to identify all FBA proteins in the potato genome. To further verify the reliability of the candidate sequences, the structural domains of the candidate FBA protein sequences were identified in the Pfam database (http://pfam.xfam.org/) and SMART database (http://smart.embl-heidelberg.de). The website Cell-Ploc 2.0 (http://www.csbo.sjtu.edu.cn/bioinf/Cell-PLoc-2) was used to predict the subcellular localization of StFBA proteins. The online software ExPASy (https://web.expasy.org/protparam/) was used to analyze the physicochemical properties of the *StFBA* members.

### 2.3 Phylogenetic, gene structure and conserved motifs analyses of StFBA

The nine StFBA protein sequences were subjected to multiple sequence alignment using ClustaW software (Thompson et al., 2002) with default parameters. Neighbor-Joining (NJ) phylogenetic tree was constructed using MEGA11 software with 1,000 bootstrap replicates. The same method was used for constructing and analyzing the phylogenetic tree of potato FBA gene family and the *FBA* genes of eight in Arabidopsis (Lu et al., 2012), eight in tomato (Cai et al., 2016), seven in eggplant, 16 in tobacco (Zhao et al., 2021), seven in rice (Zhang et al., 2016) and 21 in wheat (Lv et al., 2017). The exon-intron structures were visualized using the Gene Structure Display Server (GSDS, http://gsds.cbi.pku.edu.cn) (Hu et al., 2015). The motif prediction was performed on the MEME program (http://meme-suite.org/) (Bailey et al., 2009), with the motif length ranging from 10 to 60 amino acid residues and maximum motif number set to 20, with all other parameters being default.

### 2.4 Synteny analysis and chromosome localization

TBtools software (Chen et al., 2020) was used to obtain information such as chromosome length and gene density in the potato genome and to draw the chromosome location maps. The synteny relationships of the *FBA* genes within and among different species were calculated using MCScanX, with a threshold of *E* < 1 × 10^−5^ (Wang et al., 2012).

### 2.5 Structure prediction of StFBA proteins

SOPMA software (http://npsa-pbil.ibcp.fr/cgi-bin/npsa_automat.pl?page=npsa_sopma.html) was used to predict the secondary structure of StFBA protein. Tertiary structure prediction of StFBA proteins was performed using the latest artificial intelligence algorithm, AlphaFold2 (Jumper et al., 2021), which utilizes large-scale structural databases and deep-learning models that are able to provide more accurate protein structure prediction results. The tertiary structure visualization of StFBA proteins was performed with PyMOL2.5 software (https://pymol.org/2/).

### 2.6 Analysis of *cis*-acting elements in the promoter of *StFBA* genes

The sequence spanning 2000 bp upstream of the initiation codon of each *StFBA* gene was submitted to PlantCARE (http://bioinformatics.psb.ugent.be/webtools/plantcare/html/) (Lescot et al., 2002) for predicting *cis*-elements. TBtools software was used to draw the *cis*-acting elements in the promoter region.

### 2.7 Abiotic stress treatments of potato plants

Two batches of potato plantlets with 30 days of healthy and uniform growth were selected. The middle parts of one batch of plantlets were cut into 1–1.5 cm stem segments with leaves and inoculated into glass tubes. They were first placed in dark conditions for cultivation and after 2 days, they were exposed to blue light at a wavelength of 460 nm, red light at a wavelength of 620 nm, and white light (control) by LEDs (Opt-run Biotechnology Co., Nanjing, China). Leaves and stolon of 35 days potato plantlets and 80 days tuber under different light conditions were then collected. Another batch of potato plantlets was transferred to hydroponic conditions for 1 week and then placed in 150 mM NaCl solution and 15% polyethylene glycol (PEG6000) solution for salt and drought stress treatments at 0, 3, 6, 12, and 24 h. Then, the leaves from the top of the potato plants were collected. All tissue samples collected were immediately placed in liquid nitrogen and stored at −80°C for later use, with each treatment group containing three biological replicates.

### 2.8 Quantitative real-time PCR analysis

RNA extraction and reverse transcription were referred to Huang et al. (2017), and qRT-PCR was referred to Jin et al. (2019) method. PCR was performed using an ABIViiA7 real-time PCR instrument (Life Technologies, United States). Specific amplification primers for the *StFBA* gene were designed using NCBI Primer-BLAST (https://www.ncbi.nlm.nih.gov/) with potato *elongation factor*-*1alpha* (*EF1α*) as an internal control (Supplementary Table S4) (Tang et al., 2017). The qRT-PCR for three independent biological replicate experiments, with three replicates for each PCR reaction. The relative expression of *StFBA* genes in different tissues and different treatments was calculated using the 2^−ΔΔCT^ and 2^−ΔΔCT^ method, respectively (Livak and Schmittgen, 2001). The one-way ANOVA of variance was used to conduct the statistical analyses of data results by SPSS software, and the significance of differences was analyzed using Duncan’s Multiple Range Test (DMRT) as a *post hoc* test (*p* < 0.05). GraphPad software was used for statistical analysis and drawing of data.

## 3 Results

### 3.1 Genome-wide identification of StFBA gene family members

A total of nine *StFBA* genes were identified from the potato genome using genome-wide analysis (Table 1). The *StFBA* genes were randomly and unevenly distributed across seven chromosomes; they were named *StFBA1*∼*StFBA9* according to their order in the potato chromosome (Supplementary Figure S1). Analysis of the amino acid characteristics of StFBA family proteins showed that the number of amino acids (aa) of StFBA proteins ranged from 311 (StFBA3) to 1,379 (StFBA4), where the sequence length of the amino acid sequences of class II was significantly longer than that of class I. This difference may be due to the variations in their structure and function. The protein molecular weights ranged from 33,653.82 (StFBA3) to 148,195.01 (StFBA4) Da, while the theoretical isoelectric point varied between 5.94 (StFBA6) to 8.76 (StFBA5). Prediction of subcellular localizations of proteins showed that StFBA1∼StFBA5 and StFBA8 were localized in chloroplasts, and StFBA6, StFBA7 and StFBA9 were localized in the cytoplasm.

**TABLE 1:** Profiles of the identified FBA gene family in *S. tuberosum*.

Gene	Gene ID	Gene location	Amino acid number/aa	Molecular weight/Da	Theoretical isoelectric point	Subcellular localization
*StFBA1*	Soltu.DM.01G050210.1	86,885,838–86,892,001: +	397	42,800.77	6.85	Chloroplast
*StFBA2*	Soltu.DM.02G006160.1	19,797,274–19,799,587: −	395	42,684.59	6.39	Chloroplast
*StFBA3*	Soltu.DM.02G024280.1	37,587,897–37,590,299: −	311	33,653.82	6.76	Chloroplast
*StFBA4*	Soltu.DM.03G033020.1	56,749,621–56,770,750: +	1,379	148,195.01	5.99	Chloroplast
*StFBA5*	Soltu.DM.05G004320.1	3,719,005–3,722,380: +	395	42,910.76	8.76	Chloroplast
*StFBA6*	Soltu.DM.07G027740.1	56,783,827–56,789,828: +	357	38,216.53	5.94	Cytoplasm
*StFBA7*	Soltu.DM.09G004030.1	3,432,082–3,434,410: +	358	38,618.12	7.15	Cytoplasm
*StFBA8*	Soltu.DM.10G016600.1	46,399,698–46,402,885: +	342	37,818.92	8.25	Chloroplast
*StFBA9*	Soltu.DM.10G024820.1	56,467,722–56,471,929: +	358	38,439.96	8.52	Cytoplasm

### 3.2 Phylogeny of *StFBA* and other plant *FBA* genes

**FIGURE 1:**
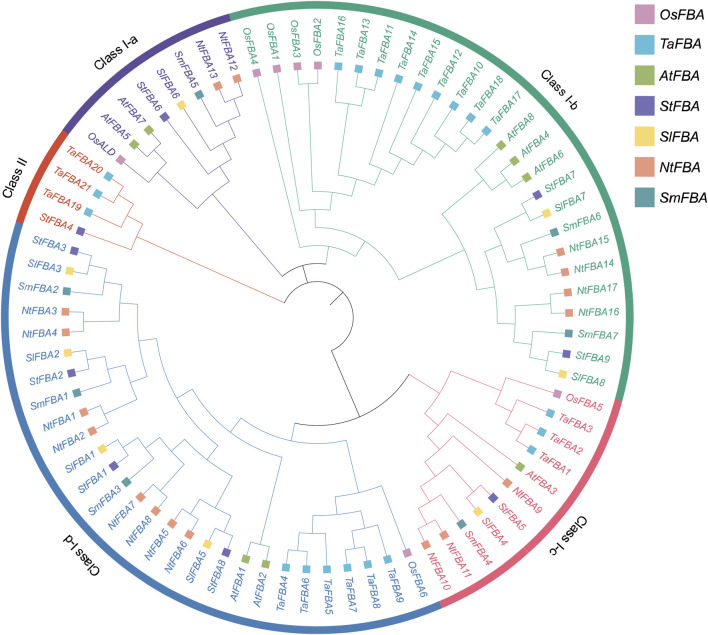
Phylogenetic tree of FBA proteins from potato and other plant species. Abbreviations represent the following species: *S. tuberosum* (*St*), *A. thaliana* (*At*), *S. lycopersicum* (*Sl*), *S. melongena* (*Sm*), *N. tabacum* (*Nt*), *Oryza sativa* (*Os*) and *Triticum aestivum* (*Ta*). Different colored squares represented different species. Different colored arcs indicate different classes or subclasses.

To determine evolutionary relationships of the FBA gene family, 76 FBA protein amino acid sequences from seven species, including *Arabidopsis*, tomato, tobacco, rice, eggplant, wheat, and potato, were used to construct a phylogenetic tree (Supplementary Table S1). The results indicated that the *StFBA* gene, like other species, was classified into class I and II subclades (Figure 1). Class I could be further divided into four subclasses. Among the nine StFBA proteins, one was in Class I-a, two in Class I-b, one in Class I-c, four in Class I-d, and one in Class II. Of these, the identified wheat *TaFBA19*-*TaFBA21* belonged to Class II, which was consistent with the clustering characteristics of *StFBA4*. The phylogenetic tree showed that FBA proteins were more closely related and similar between potato and other *Solanaceae* plants. In terms of evolutionary relationships, the FBA genes of potato and tomato were more closely related than those of other species, suggesting that they might have similar biological functions.

### 3.3 Gene structure and protein motif analyses of the StFBA gene family

**FIGURE 2:**
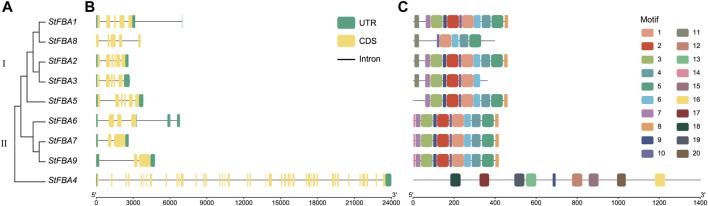
Phylogenetic relationships, gene structure, and conserved motifs of the *StFBA* gene family: **(A)** Phylogenetic relationships of the nine *StFBA* genes. **(B)** Gene structures of *StFBA*. UTR, untranslated region; CDS, coding sequence. **(C)** Distributions of the conserved motifs within *StFBA* proteins.

Phylogenetic analysis indicated that *FBA* was divided into two classes in potatoes. Eight *StFBA* genes were identified in Class I and only *StFBA4* was identified in Class II (Figure 2A). Analyses of gene structures revealed significant variations in exon count and sequence length among different subgroups of *StFBA* genes (Figure 2B). Class I had 3–6 exons, with the fewest exons found in *StFBA7* and *StFBA9*, and the most in *StFBA2* and *StFBA5*. Class II contained 42 exons, which is significantly more than Class I members, suggesting that *StFBA4* might have a more complex structure, potentially including functional domains or regulatory elements. The motif distributions in FBA proteins were analyzed, and 20 conserved motifs were predicted (Supplementary Figure S2). Motif 1, motif 5, motif 6, and motif 10 were exclusive to Class I. StFBA1 and StFBA2 had 12 common motifs. StFBA6, StFBA7, and StFBA9 had 11 common motifs, with motif3 is a conserved motif specific to them. Moreover, some genes are missing more motifs, for example, StFBA8 contains only 6 motifs, suggesting that its function may be altered. Except for motif 9, the remaining eight motifs were specific to StFBA4 in Class II (Figure 2C). Overall, the type and distribution of conservative base sequences of Class I StFBA showed a high degree of uniformity, indicating that the structures of StFBA proteins were highly conserved in specific subclasses. In contrast, the gene structure and function of members of the potato FBA gene family might be varied between subclasses.

### 3.4 Tertiary structure of the StFBA proteins

**FIGURE 3:**
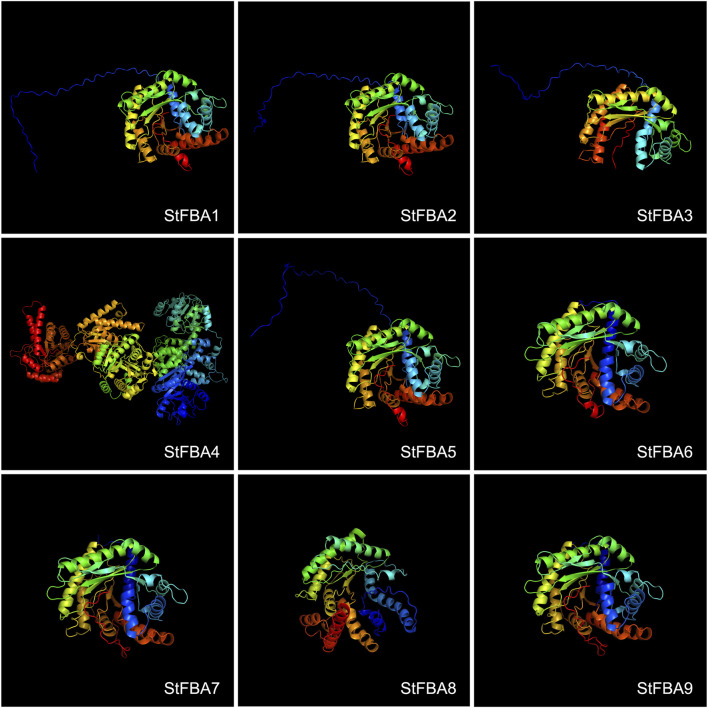
Predicted tertiary structures of FBA proteins in *S. tuberosum*.

Analyses of tertiary structures revealed that the StFBA proteins mainly consisted of α-helices, β-turns, irregular coils, and extended chains. The percentage of α-helix was the highest (42.77%–53.22%), followed by irregular coils (27.17%–33.76%), while extended chains and β-folds accounted for lower percentages (Supplementary Table S2). Comparison of the predicted tertiary structures of StFBA proteins revealed significant differences in the protein structures of different subclasses. The three-dimensional conformations of eight Class I *StFBA* gene family members were comparable (Figure 3). StFBA1, StFBA2, and StFBA5 were predicted to have more consistent protein structures. Compared to other members, the StFBA3 protein had a simpler structure, with fewer α-helices and β-turns, implying possible changes in its function. The remaining members had subtle differences in protein structures. StFBA4 had a larger number of amino acid sequences and more complex protein tertiary structures, suggesting that it might be involved in more intricate biological functions.

### 3.5 Synteny analysis of *FBA* gene family members

**FIGURE 4:**
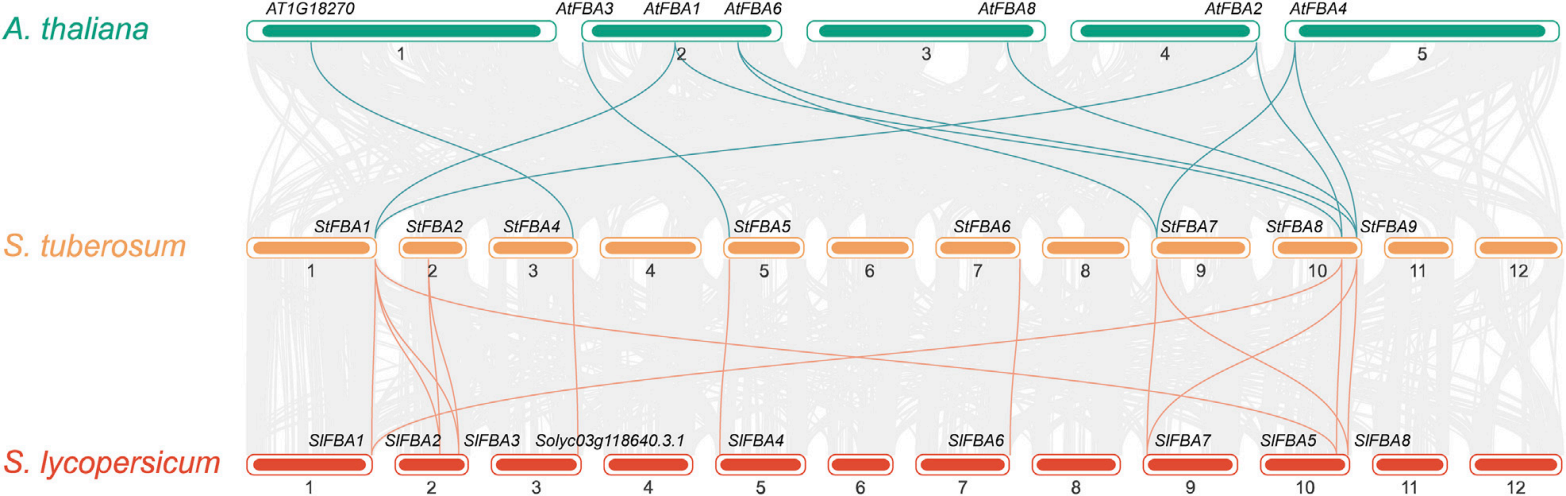
Collinear relationships of *FBA* genes in *S. tuberosum*, *A. thaliana*, and *S. lycopersicum*. The gray lines in the background indicate collinearity between potato genes and those of other species, and the green and yellow lines indicate collinear *FBA* gene pairs.

To study the contraction and expansion of *StFBA* gene family members during evolution, the *FBA* genes of potato, tomato, and *Arabidopsis* were compared using collinearity analyses (Figure 4). The results showed that 11 *StFBA* members were found to have synteny with *AtFBA*, but *StFBA2*, *StFBA3,* and *StFBA6* had no homologous genes in *Arabidopsis*. There were 14 pairs of syntenic *FBA* genes between potatoes and tomatoes, suggesting that the *FBA* genes were more homologous and conserved in Solanaceae than in dicotyledons. It is noteworthy that we have found that *StFBA4* is homologous to *AT1G18270* in *Arabidopsis* and *Solyc03g118640.3.1* in tomato. Although *AT1G18270* (NCBI Reference Sequence: NP_001117303.1) is annotated as ketose-bisphosphate aldolase class-II family protein, and *Solyc03g118640.3.1* (NCBI Reference Sequence: XP_004235744.1) is annotated as uncharacterized protein LOC101261901. They all contain fructose-bisphosphate aldolase class-II (PF01116) domain and is further assigned as fructose-bisphosphate aldolase. Thus, AT1G18270 and Solyc03g118640.3.1 are actually both fructose-bisphosphate aldolase class II family proteins. Additionally, there were four pairs of duplicate segments, *StFBA1*-*StFBA2*, *StFBA2*-*StFBA3*, *StFBA1*-*StFBA3,* and *StFBA1*-S*tFBA8,* in the potato genome region (Supplementary Figure S3). Expansion of potato *FBA* family members was mainly associated with segmental duplication events (Supplementary Table S3).

### 3.6 Expression patterns of *StFBA* genes in different tissues

**FIGURE 5:**
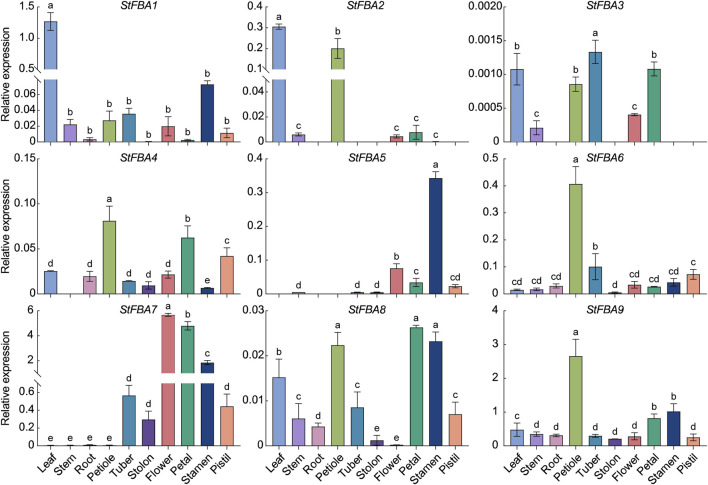
Expression analyses of *StFBA* genes in different tissues. Significant differences among the groups were compared based on Duncan’s test (*p* < 0.05). The data points represent the mean ± SD.

To further understand the potential function of *StFBA* genes, the expression levels of nine *StFBA* genes in a variety of tissues were analyzed (Figure 5). It was found that some class I *StFBA* genes showed tissue specificities. For example, *StFBA1* was highly expressed in leaves, *StFBA2* was mainly expressed in green organs compared to other tissues, while *StFBA5* was mainly expressed in floral organs. *StFBA6* and *StFBA9* were specifically expressed in petiole and had similar expression characteristics. *StFBA3* was expressed at a lower level and was not expressed in many tissues. Most of the *FBA* genes were expressed in all tissues, indicating that they might be involved in the growth and development of potatoes. Specifically, *StFBA7* was highly expressed in floral organs, tubers, and stolon, suggesting it might serve a pivotal function during flowering and tuber formation in potatoes. The class II *StFBA4* was expressed in most tissues, but at a lower expression level.

### 3.7 Expression patterns of *StFBA* genes in response to different light

**FIGURE 6:**
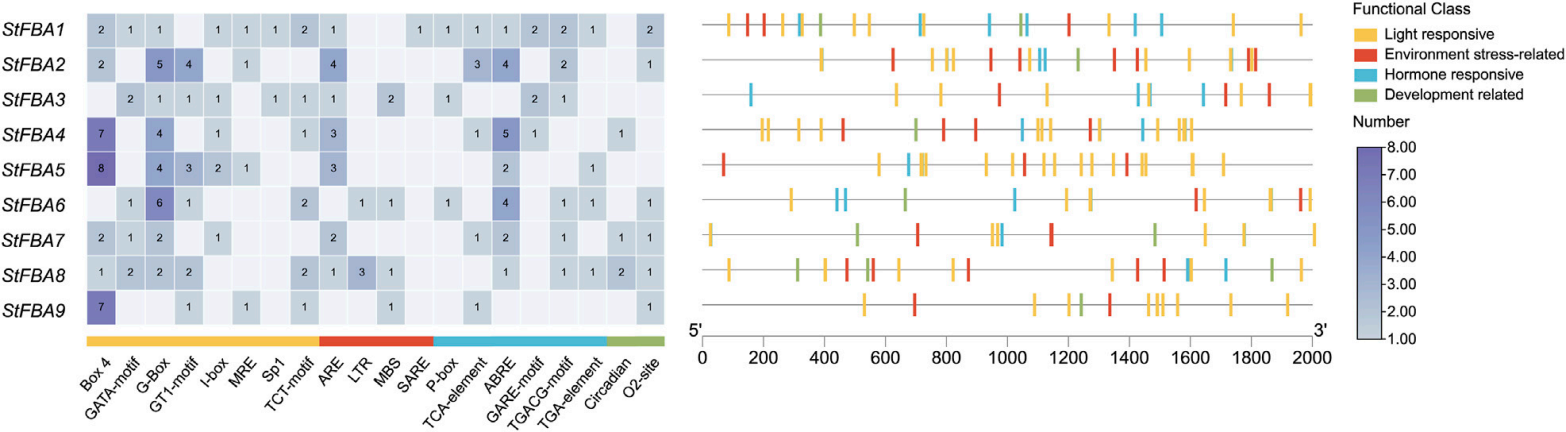
Distribution of *cis*-elements in the promoter regions of *StFBA* genes. The heat map represents classifications and statistics of *cis*-acting components, the *cis*-elements were divided into four broad categories. Different colors represent different types of elements, and the ruler at the bottom indicates the direction and length of the sequence.

**FIGURE 7:**
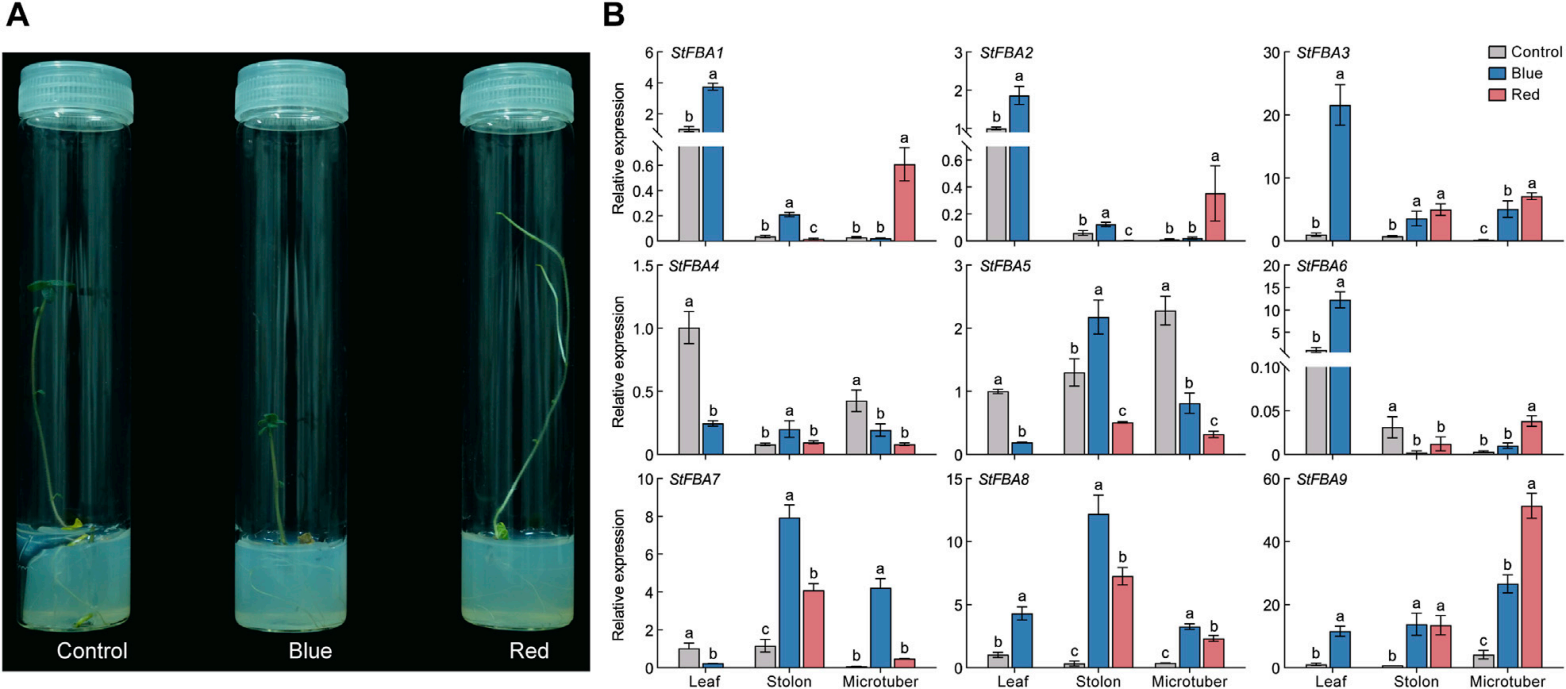
Growth of potato plantlet under different light spectrum and expressions of nine *StFBA* genes in different tissues under red and blue light treatments: **(A)** Growth of potato plantlet under different light spectrum. Blue, 460 nm blue light; red, 620 nm red light; control, white light. **(B)** The qRT-PCR was performed to analyze the relative expression levels of *StFBA* genes. Error bars represent standard deviations of the means from three independent experiments. Significant differences among the groups were compared based on Duncan’s test (*p* < 0.05). The data points represented the mean ± SD.

Multiple *cis*-regulatory elements were detected in the promoter region of *StFBA* genes (Figure 6). Based on functions of the *cis*-acting elements, the selected 20 elements were categorized into four groups: light response, stress response, hormone response and plant growth and development. Hormonal response elements included responses to auxin, abscisic acid, gibberellin, and salicylic acid; and stress included responses to drought and cold stress elements. We found that multiple light-responsive elements existed in the promoter regions of each *StFBA* gene, indicating that the *StFBA* genes were an important component of light responses in potatoes. Thus, the expression levels of *StFBA* in leaves, stolon, and microtubers were analyzed under different light treatments. The expression patterns of *FBA* genes exhibited discrepancies under different spectra. Due to monochromatic red light exposure, the leaves development of the microtubers was severely inhibited, hence there was no leaf sample under red light (Figure 7A). Under blue light, *StFBA7* showed lower expression in leaves, but significantly increased expression in stolon and microtubers. Under red light, *StFBA3*, *StFBA7*, *StFBA8,* and *StFBA9* showed higher expressions, when compared to under white light in stolon. In contrast, the expression levels of *StFBA1*, *StFBA2*, *StFBA3,* and *StFBA9* were significantly higher than that of the other treatments in tubers. Class II *StFBA4* gene showed lower expression in all tissues under red and blue light, suggesting that the gene might be spectrally insensitive. Moreover, the expression levels of *StFBA1* and *StFBA2* in all tissues were highly similar under various spectral treatments, suggesting that these genes might have similar regulatory mechanisms in response to different lighting conditions (Figure 7B).

### 3.8 Expression profiles of the *StFBA* genes in response to abiotic stresses

**FIGURE 8:**
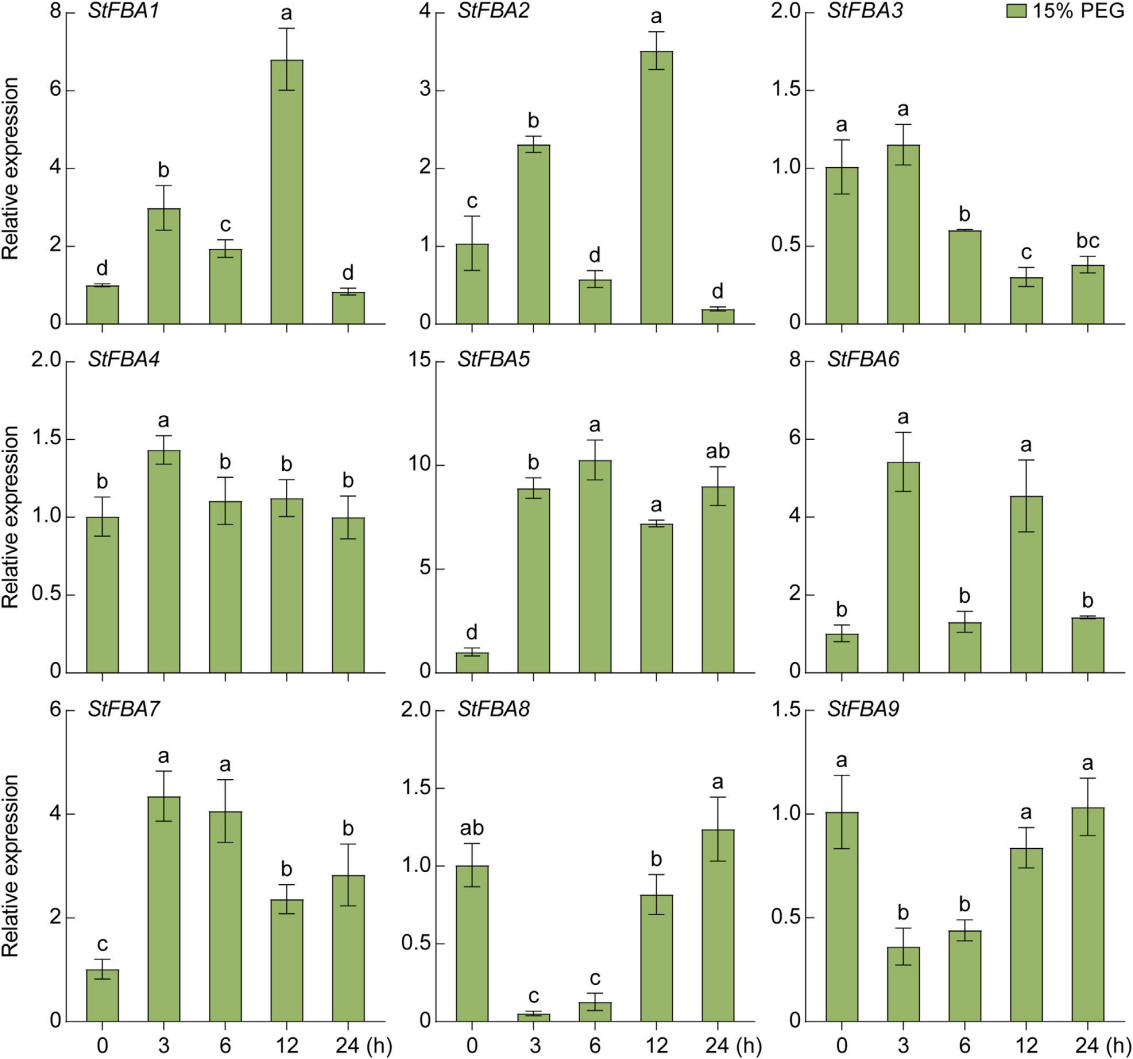
Expression profiles of the *StFBA* genes after 15% PEG6000 simulated drought stress. PEG, polyethylene glycol. Significant differences among the groups were compared based on Duncan’s test (*p* < 0.05). The data points represented the mean ± SD.

**FIGURE 9:**
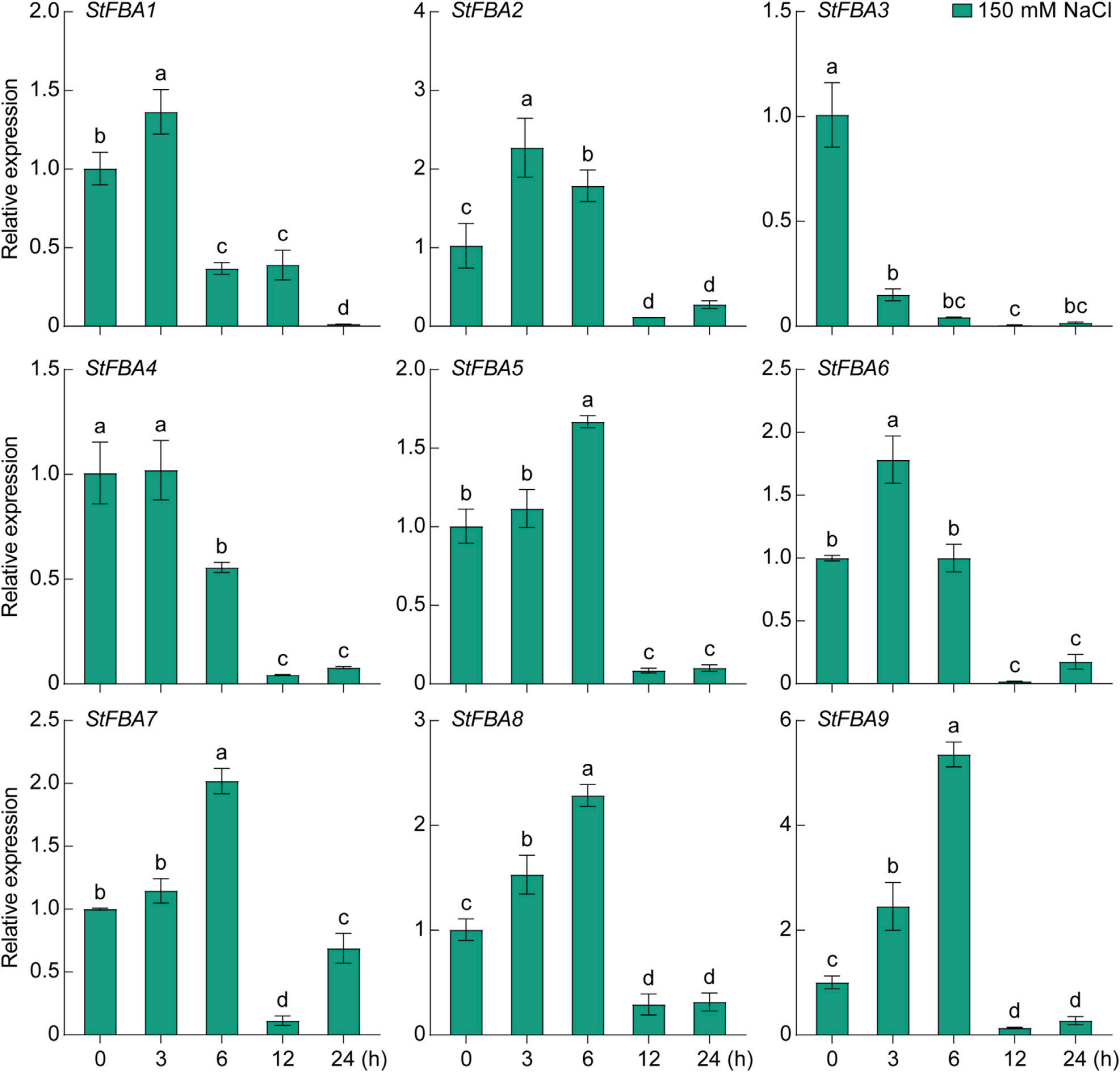
Expression profiles of the *StFBA* genes under salt stress treatment using 150 mM NaCl. Significant differences among the groups were compared based on Duncan’s test (*p* < 0.05). The data points represent the mean ± SD.

The expression patterns of the *StFBA* gene ins response to abiotic stress such as drought and salt were determined, indicating that the expression levels of the nine *StFBA* genes varied to different degrees under different stress treatments. Under the 15% PEG6000 treatment, *StFBA3* mainly showed a decreasing trend, while expression levels of *StFBA5* and *StFBA7* were significantly upregulated, so they might be important candidate genes involved in drought stress. *StFBA4* expression was minimally affected. The expression levels of *StFBA5* and *StFBA7* were significantly upregulated (*p* < 0.01). The expression patterns of the remaining genes were more complicated. For example, *StFBA1, StFBA2,* and *StFBA6* were highly induced at 3 h, then decreased at 6 h, then finally increased again at 12 h treatment, *StFBA8* and *StFBA9* expression levels showed a tendency to decrease and then increase, and all of these genes gradually recovered to their untreated expression levels at 24 h (Figure 8). Under salt conditions (150 mM NaCl), expression of *StFBA3* significantly decreased. The expressions of *StFBA1*, *StFBA2*, *StFBA4,* and *StFBA6* peaked at 3 h, while the expressions of *StFBA5*, *StFBA7*, *StFBA8,* and *StFBA9* peaked at 6 h, then all of them showed a decreasing trend (Figure 9). Together, the results further confirmed the possible role of the StFBA gene family in potato responses to various abiotic stresses.

## 4 Discussion

Organic compounds produced by photosynthesis can promote the formation and enlargement of potatoes (Fernie and Willmitzer, 2001). As a key enzyme in the Calvin cycle and glycolysis, *FBA* participates in the metabolic process of starch biosynthesis. Decreasing the activity of this enzyme can result in stunted potato growth, reduced photosynthetic efficiency, and an impact on tuber starch content, ultimately affecting potato yield and quality (Kossmann et al., 1995). Hence, *FBA* plays a vital regulatory role in the growth and tuber yield of potatoes. Our study identified nine *FBA* genes in the complete genome sequence of potatoes, which were divided into Class I and Class II members, based on their two structural domains (Table 1). Potatoes, like *Arabidopsis* and tomatoes, possess eight Class I members, indicating the conservation of Class I *StFBA* genes during their evolution. Class I and Class II *FBA* have no similarities in gene sequence, protein structure, and function, and are often considered to have evolved from different origins (Schnarrenberger et al., 1990; Zgiby et al., 2000). We identified one Class II *FBA* gene in *S*. *tuberosum*, which has 1,379 amino acids and 42 exons, a significantly higher number than Class I. This finding aligns with observations in other plant species, three have been identified in *Triticum aestivum* (Lv et al., 2017), two in *B*. *napus* (Zhao et al., 2019), and four in cotton (Li et al., 2021). The amino acid numbers of these genes ranged from 1,352 to 1,383, with exon numbers between 41 and 42. This indicates that Class II FBAs are conserved among different species and might have similar functions. According to the conserved motif analysis results of StFBA, we found that apart from StFBA1 and StFBA2, which contain all 10 motifs, other members exhibited a certain degree of motif loss, with the most loss in StFBA8, suggesting possible functional changes in this gene. Class II FBA shared fewer motifs in common with Class I (Figure 2). Overall, members within the same subclass showed similarities in sequence, gene structure, and conserved motif composition, indicating that *StFBA* family members may have undergone similar genetic evolution processes.

Based on the phylogenetic tree, eight Class I *StFBA* members were further divided into four subclasses (Figure 1), the Class I-d subclass had the most clustered *StFBA* genes. Closely related gene members in the phylogenetic tree may have similar structures and functions. Because both *S*. *tuberosum* and *S*. *lycopersicum* belong to the *Solanaceae* plant family, they were closer in the evolutionary tree. It is worth noting that *SlFBA7* and *StFBA7* clustered together, had the same number of amino acids, and were located in the cytoplasm. Overexpression of *SlFBA7* can significantly increase the *FBA* activity and the expression level of other enzymes in the Calvin cycle of tomato seedling leaves, along with an increase in regeneration of RuBP (Cai et al., 2016). Therefore, we speculate that *StFBA7* may play a pivotal role in enhancing the photosynthetic efficiency of potatoes. At present, the functions of some *FBA* genes have been thoroughly studied in model plants such as *A*. *thaliana* and *O*. *sativa* (Lu et al., 2012; Simkin et al., 2017). For example, *AtFBA2* has the highest activity and expression level in leaves, and overexpression of *AtFBA2* boosts the photosynthetic capacity and biomass of the plants. However, *fba2* mutant plants are dwarfed and the starch level is significantly reduced. *AtFBA3* is mainly expressed in the vascular tissues of leaves and roots, so the absence of *AtFBA3* limits the biosynthesis of essential compounds such as amino acids. Similarly, the *fba3* mutants grow more slowly and show a decrease in photosynthesis (Carrera et al., 2021).

Collinearity analysis between *S*. *tuberosum* and *A*. *thaliana* revealed that *AtFBA2* was homologous with *StFBA1* and *StFBA8*, while *AtFBA3* shared homology with *StFBA5* (Figure 4). We speculate that *StFBA1*, *StFBA5,* and *StFBA8* might contribute to enhancing tuber yield. However, their specific functions and mechanisms of action still require further confirmation. In plants, gene duplication events primarily consist of tandem duplication and segmental duplication, which play key roles in biological evolution, gene family formation, and the transmission of genetic information (Kong et al., 2007; Paterson et al., 2010). The members of the *StFBA* gene family were closely associated with segmental duplication events. The multiple occurrences of *StFBA1* in repetitive events suggested that this gene may have played a significant role in driving the evolution of the FBA gene family, leading to gene amplifications and functional changes. The tertiary structure of proteins determines their function in cells and how they interact with other molecules (Kuhlman and Bradley, 2019). Based on the physicochemical properties and predicted protein structure of StFBA, we showed that the structures of StFBA3 and StFBA8 were simpler than other members (Figure 3). These two proteins exhibited the lowest number of amino acids and the smallest percentage of α-helices (Supplementary Table S2). It is therefore possible that during the process of segmental duplication, these two genes underwent loss or alterations in some of their conserved domains and functions. *StFBA6*, *StFBA7,* and *StFBA9* were located in the cytoplasm, had very similar amino acid numbers, and showed a high degree of similarity in their secondary and tertiary structures, indicating that they may be homologous genes.

Through the analysis of transcriptome data and qRT-PCR validation, we obtained the expression levels of *StFBA* genes in different potato tissues. *StFBA7* exhibited higher expressions in tubers, stolon, and flower organs, indicating that it may be a key gene regulating the processes of microtuber formation and expansion (Figure 5). The expression patterns of *StFBA* family members in different tissues demonstrated that *FBA* genes had specific roles during potato growth and development. The *cis*-acting element analysis revealed an abundance of light-responsive elements within the StFBA gene family (Figure 6). Throughout the growth and development of potatoes, light influenced tuber induction and expansion via the processes of the photoperiod, light intensity, and spectrum. Among them, light spectrum is one of the key signals regulating tuberization in potato plants. It has been reported that the transcription level of StBEL5, a primary signal for potato tuberization, was mostly affected by the light spectrum, with the red-blue spectrum inducing the initiation of StBEL5 synthesis (Chatterjee et al., 2007). Further studies found that blue light at 460 nm wavelength aided tuber expansion, while 620 nm red light was more favorable for tuber induction (Li et al., 2020). The microtubers under blue light were significantly larger than other spectra in our study (Supplementary Figure S4). The qRT-PCR results indicated that *StFBA* genes were highly responsive to spectral changes, with *StFBA3*, *StFBA8,* and *StFBA9* showing significantly higher expression levels in leaf, stolon, and tuber, when compared to the white light control. These results implied that they may improve photosynthesis and play an important function in regulating the induction and expansion of microtubers (Figure 7).

Potato is highly sensitive to drought and salt stress. Drought affects the aboveground plant height and leaf area index, leading to a reduction in photosynthetic efficiency and adversely affecting potato yield and quality through shortened growth cycles and decreased underground tuber quantity and size (Deblonde and Jean-François, 2001). High salt stress disrupts the source-sink balance in potatoes, impairing the production of antioxidants and antioxidant enzyme activity, and accelerating the occurrence of plant diseases (Albacete et al., 2014; Chourasia et al., 2021). Studies have shown that *FBA* genes played a role in responses to various abiotic stresses, including drought, high salt (Yamada et al., 2000), and cold stress (Liu et al., 2019). We found that under drought stress, the expression of *StFBA5* significantly increased, and it maintained a high expression level even after 24 h (Figure 8). When exposed to high salt stress, the expression levels of most *StFBA* genes showed an initial increase followed by a sharp decline (Figure 9). These results indicated that *StFBA* members played a crucial role in responding to different environmental stress. In addition, *FBA* has been found to have a wide range of functions in other species. For example, *AtFBA6* participates in the memory of high temperature stress in the apical meristem of *Arabidopsis* (Olas et al., 2021). *AtFBA8* responds to environmental humidity changes and interacts with actin, involving guard cell opening and closing (Van der Linde et al., 2011). The soybean *gmfbacgmfbac2* double mutant exhibits slow growth, narrowed leaves, dwarfed seedlings, and severe imbalance in carbon-nitrogen metabolism (Qiu et al., 2023). It is hypothesized that the *StFBA* gene may have functional diversity, but the specific functional mechanism needs to be verified through future experiments.

## 5 Conclusion

In this study, eight class I and one class II *StFBA* gene members were identified using genome-wide analyses. We found that the *FBA* of potatoes and tomatoes were closely related in the evolutionary tree and in synteny relationships. *StFBA1* was a key gene in the fragment duplication event within the gene family. *StFBA6*, *StFBA7,* and *StFBA9* were involved in growth and development of potatoes. *StFBA3*, *StFBA8,* and *StFBA9* may influence photosynthesis and play a critical function in regulating the induction and expansion of microtubers, while *StFBA5* and *StFBA9* played essential roles in responses to drought and salt stress. The results of this study provided a basis for understanding the potential functions of the StFBA gene family, and presented ideas for the improvement of photosynthetic efficiency and response to adversity stress of potatoes.

## Data Availability

The datasets presented in this study can be found in online repositories. The names of the repository/repositories and accession number(s) can be found in the article/Supplementary Material.
