# Supplementary material for: Characterization of FBA genes in potato (Solanum tuberosum L.) and expression patterns in response to light spectrum and abiotic stress
Source: Front Genet. 2024 Apr 12;15:1364944. doi: 10.3389/fgene.2024.1364944 (PMC11057440; doi:10.3389/fgene.2024.1364944)
Supplement: Supplementary file 1 [file DataSheet1.ZIP › Figures and Tables Captions.docx]

**Table 1**. Information of the identified FBA gene family in S. *tuberosum*.

**Figure 1**. Phylogenetic tree of FBA proteins from potato and other plant species. Abbreviations represent the following species: *S*. *tuberosum* (*St*), *A*. *thaliana* (*At*), *S*. *lycopersicum* (*Sl*), *S*. *melongena* (*Sm*), *N*. *tabacum* (*Nt*), *Oryza sativa* (*Os*) and *Triticum aestivum* (*Ta*). Different colored squares represented different species. Different colored arcs indicate different classes or subclasses.

**Figure 2**. Phylogenetic relationships, gene structure, and conserved motifs of the *StFBA* gene family: (**A**): Phylogenetic relationships of the nine *StFBA* genes. (**B**): Gene structures of *StFBA*. UTR, untranslated region; CDS, coding sequence. (**C**): Distributions of the conserved motifs within StFBA proteins.

**Figure 3**. Predicted tertiary structures of FBA proteins in S. *tuberosum.*

**Figure 4**. Collinear relationships of *FBA* genes in *S*. *tuberosum*, *A*. *thaliana,* and *S*. *lycopersicum*. The gray lines in the background indicate collinearity between potato genes and those of other species, and the green and yellow lines indicate collinear *FBA* gene pairs.

**Figure 5**. Expression analyses of *StFBA* genes in different tissues. Significant differences among the groups were compared based on Duncan’s test (*P* < 0.05). The data points represent the mean ± SD.

**Figure 6**. Distribution of *cis*-elements in the promoter regions of *StFBA* genes. The heat map represents classifications and statistics of *cis*-acting components, the *cis*-elements were divided into four broad categories. Different colors represent different types of elements, and the ruler at the bottom indicates the direction and length of the sequence.

**Figure 7**. Growth of potato plantlet under different light spectrum and expressions of nine *StFBA* genes in different tissues under red and blue light treatments: (**A**): Growth of potato plantlet under different light spectrum. Blue, 460 nm blue light; red, 620 nm red light; control, white light. (**B**): The qRT-PCR was performed to analyze the relative expression levels of *StFBA* genes. Error bars represent standard deviations of the means from three independent experiments. Significant differences among the groups were compared based on Duncan’s test (*P* < 0.05). The data points represented the mean ± SD.

**Figure 8**. Expression profiles of the *StFBA* genes after 15% PEG6000 simulated drought stress. PEG, polyethylene glycol. Significant differences among the groups were compared based on Duncan’s test (*P* < 0.05). The data points represented the mean ± SD.

**Figure 9.** Expression profiles of the *StFBA* genes under salt stress treatment using 150 mM NaCl. Significant differences among the groups were compared based on Duncan’s test (*P* < 0.05). The data points represent the mean ± SD.
