# Supplementary material for: Characterization of FBA genes in potato (Solanum tuberosum L.) and expression patterns in response to light spectrum and abiotic stress
Source: Front Genet. 2024 Apr 12;15:1364944. doi: 10.3389/fgene.2024.1364944 (PMC11057440; doi:10.3389/fgene.2024.1364944)

Supplementary Material

Genome-wide identification and characterization analysis of *FBA* genes in Potato (*Solanum tuberosum* L.) and expression patterns in response to light spectrum and abiotic stress

Ting Li^1†^, Xinyue Hou^1†^, Zhanglun Sun^1^, Bin Ma^2^, Xingxing Wu^1^, Tingting Feng^1^, Hao Ai^1^, Xianzhong Huang^1*^ and Ruining Li^1*^

*Correspondence: Xianzhong Huang: huangxz@ahstu.edu.cn; Ruining Li: lirn@ahstu.edu.cn

# Supplementary Table

**Supplementary Table 1**. FBA gene family profiles of different species

| **Gene Name** | | **Gene ID** | **Amino acid number/aa** | | **Subcellular localization** | |
| --- | --- | --- | --- | --- | --- | --- |
| *ALDY* | LOC Os06g40640.1 | | | 358 | | Cytoplasm |
| *OsFBA1* | LOC_Os01g67860.1 | | | 358 | | Cytoplasm |
| *OsFBA2* | LOC_Os05g33380.1 | | | 358 | | Cytoplasm |
| *OsFBA3* | LOC_Os10g08022.1 | | | 358 | | Cytoplasm |
| *OsFBA4* | LOC_Os08g02700.1 | | | 362 | | Cytoplasm |
| *OsFBA5* | LOC_Os01g02880.1 | | | 388 | | Chloroplast |
| *OsFBA6* | LOC_Os11g07020.1 | | | 388 | | Chloroplast |
| *TaFBA1* | LOC123059824 | | | 387 | | Chloroplast |
| *TaFBA2* | LOC542930 | | | 385 | | Chloroplast |
| *TaFBA3* | LOC123076846 | | | 387 | | Chloroplast |
| *TaFBA4* | LOC123086572 | | | 388 | | Chloroplast |
| *TaFBA5* | LOC123091418 | | | 388 | | Chloroplast |
| *TaFBA6* | LOC100415821 | | | 388 | | Chloroplast |
| *TaFBA7* | LOC123102769 | | | 385 | | Chloroplast |
| *TaFBA8* | LOC123111000 | | | 385 | | Chloroplast |
| *TaFBA9* | LOC123120002 | | | 385 | | Chloroplast |
| *TaFBA10* | LOC123062806 | | | 358 | | Cytoplasm |
| *TaFBA11* | LOC123062809 | | | 358 | | Cytoplasm |
| *TaFBA12* | LOC123071650 | | | 358 | | Cytoplasm |
| *TaFBA13* | LOC123071651 | | | 358 | | Cytoplasm |
| *TaFBA14* | LOC123079989 | | | 358 | | Cytoplasm |
| *TaFBA15* | LOC123079984 | | | 358 | | Cytoplasm |
| *TaFBA16* | LOC123079991 | | | 358 | | Cytoplasm |
| *TaFBA17* | gnl\|TA_TGACv1.30.dna.genome\|  TGACv1_scaffol d_394081_5AS  dna: scaffold: 1:45711:1 | | | 244 | | Cytoplasm |
| *TaFBA18* | TGACv1_scaffold_423736_5BS | | | 519 | | Cytoplasm |
| *TaFBA19* | LOC123149877 | | | 1383 | | Chloroplast |
| *TaFBA20* | LOC123160810 | | | 1383 | | Chloroplast |
| *TaFBA21* | LOC123169616 | | | 1383 | | Chloroplast |
| *AtFBA1* | AT2G21330 | | | 399 | | Chloroplast |
| *AtFBA2* | At4G38970 | | | 398 | | Chloroplast |
| *AtFBA3* | AT2G01140 | | | 391 | | Chloroplast |
| *AtFBA4* | AT5G03690 | | | 393 | | Mitochondrion |
| *AtFBA5* | AT4G26530 | | | 358 | | Cytoplasm |
| *AtFBA6* | AT2G36460 | | | 358 | | Cytoplasm |
| *AtFBA7* | AT4G26520 | | | 358 | | Cytoplasm |
| *AtFBA8* | AT3G52930 | | | 358 | | Cytoplasm |
| *SlFBA1* | Solyc01g110360.2.1 | | | 397 | | Chloroplast |
| *SlFBA2* | Solyc02g062340.2.1 | | | 395 | | Chloroplast |
| *SlFBA3* | Solyc02g084440.2.1 | | | 392 | | Chloroplast |
| *SlFBA4* | Solyc05g008600.2.1 | | | 395 | | Chloroplast |
| *SlFBA5* | Solyc10g054390.1.1 | | | 136 | | Chloroplast |
| *SlFBA6* | Solyc07g065900.2.1 | | | 431 | | Cytoplasm |
| *SlFBA7* | Solyc09g009260.2.1 | | | 358 | | Cytoplasm |
| *SlFBA8* | Solyc10g083570.1.1 | | | 358 | | Cytoplasm |
| *NtFBA1* | gene_57322 | | | 397 | | Chloroplast |
| *NtFBA2* | gene_38021 | | | 397 | | Chloroplast |
| *NtFBA3* | gene_60848 | | | 398 | | Chloroplast |
| *NtFBA4* | gene_73919 | | | 398 | | Chloroplast |
| *NtFBA5* | gene_62167 | | | 395 | | Chloroplast |
| *NtFBA6* | gene_35220 | | | 246 | | Chloroplast |
| *NtFBA7* | gene_68314 | | | 396 | | Chloroplast |
| *NtFBA8* | gene_3622 | | | 396 | | Chloroplast |
| *NtFBA9* | gene_54243 | | | 395 | | Chloroplast |
| *NtFBA10* | gene_20790 | | | 226 | | Chloroplast |
| *NtFBA11* | gene_38975 | | | 358 | | Cytoplasm |
| *NtFBA12* | gene_22224 | | | 358 | | Cytoplasm |
| *NtFBA13* | gene_67031 | | | 357 | | Cytoplasm |
| *NtFBA14* | gene_41189 | | | 357 | | Cytoplasm |
| *NtFBA15* | gene_76667 | | | 358 | | Cytoplasm |
| *NtFBA16* | gene_37698 | | | 358 | | Cytoplasm |
| *SmFBA1* | Smechr0100179.1 | | | 395 | | Chloroplast |
| *SmFBA2* | Smechr0200681.1 | | | 395 | | Chloroplast |
| *SmFBA3* | Smechr0202275.1 | | | 395 | | Chloroplast |
| *SmFBA4* | Smechr0702691.1 | | | 357 | | Mitochondrion |
| *SmFBA5* | Smechr0900105.1 | | | 358 | | Cytoplasm |
| *SmFBA6* | Smechr1000050.1 | | | 392 | | Chloroplast |
| *SmFBA7* | Smechr1002525.1 | | | 358 | | Mitochondrion |

**Supplementary Table 2**. Prediction of secondary structure of potato FBA protein

| **Name** | **Alpha helix** | **Beta turn** | **Random coil** | **Extended strand** |
| --- | --- | --- | --- | --- |
| StFBA1 | 49.62% | 6.55% | 29.72% | 14.11% |
| StFBA2 | 45.82% | 6.08% | 33.16% | 14.94% |
| StFBA3 | 42.77% | 7.07% | 33.76% | 16.40% |
| StFBA4 | 46.48% | 8.27% | 29.01% | 16.24% |
| StFBA5 | 47.59% | 6.84% | 30.63% | 14.94% |
| StFBA6 | 53.22% | 7.28% | 27.17% | 12.32% |
| StFBA7 | 51.68% | 5.87% | 29.05% | 13.41% |
| StFBA8 | 49.42% | 5.26% | 30.41% | 14.91% |
| StFBA9 | 51.12% | 7.26% | 28.21% | 13.41% |

| **Supplementary Table 3**. Gene duplication of *FBA* gene in *Solanum tuberosum* | | | | |
| --- | --- | --- | --- | --- |
| **Gene Name** | **Gene ID** | **Gene Name** | **Gene ID** | **Duplication type** |
| *StFBA1* | Soltu.DM.01G050210.1.v6.1 | *StFBA2* | Soltu.DM.02G006160.1.v6.1 | WGD or Segmental |
| *StFBA2* | Soltu.DM.02G006160.1.v6.1 | *StFBA3* | Soltu.DM.02G024280.2.v6.1 | WGD or Segmental |
| *StFBA3* | Soltu.DM.02G024280.2.v6.1 | *StFBA1* | Soltu.DM.01G050210.1.v6.1 | WGD or Segmental |
| *StFBA8* | Soltu.DM.10G016600.1.v6.1 | *StFBA1* | Soltu.DM.01G050210.1.v6.1 | WGD or Segmental |

**Supplementary Table 4**. Information of primer sequence

| **Gene** | **Primer sequence (5′→3′)** |
| --- | --- |
| *StFBA1* | *GTCTACCGTTGATGGACGCA* |
|  | *TGGTAGTATGCAGCAGAGCG* |
| *StFBA2* | *TAAAAGGTCAGGCACTCCGC* |
|  | *GATGCAACAGTTTTCGCGGT* |
| *StFBA3* | *CTTTGAAGTCGCCAAGCAGG* |
|  | *GTCCACCAGACAAAAATTGCC* |
| *StFBA4* | *TGGCGCACTCGAAAAAGATG* |
|  | *ACACAAGCCATAGAGGTCCTTC* |
| *StFBA5* | *GCAACTGCCGGAAAGAGACTG* |
|  | *TAGGGGAACCAAACCCTTGTC* |
| *StFBA6* | *AAAGCAGGTGCTCGTTTTGC* |
|  | *GGCTCCACAATTGGCACAAG* |
| *StFBA7* | *GTACCCATTGTTGAGCCCGA* |
|  | *GAACAGCAGGTGGCATTGTG* |
| *StFBA8* | *GTGGCACATGTCCTTCTCCT* |
|  | *AAGGCCTTCTTCGGTGTACT* |
| *StFBA9* | *CGACAAGGGTACCGTAGAG* |
|  | *TCAGGCTCAACAATGGGGAC* |
| *EF-1-alpha* | *GATGGTCAGACCCGTGAACA* |
|  | *CCTTGGAGTACTTCGGGGTG* |

# Supplementary Figures

**Supplementary Figure S1**. Chromosome distribution of *FBA* genes in S. *tuberosum*. The left scale indicates chromosome length. The inner circle indicates the number of chromosomes in the potato genome. Blue to red on the chromosome indicates gene density.


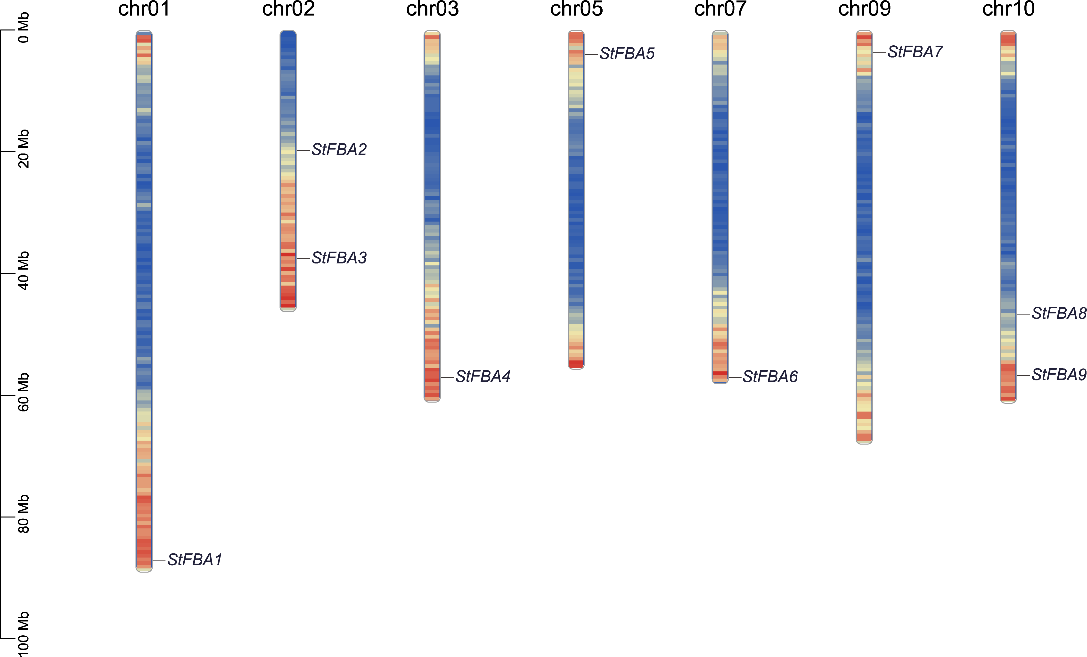


**Supplementary Figure S2**. The sequence logo conserved motif of the potato FBA proteins.
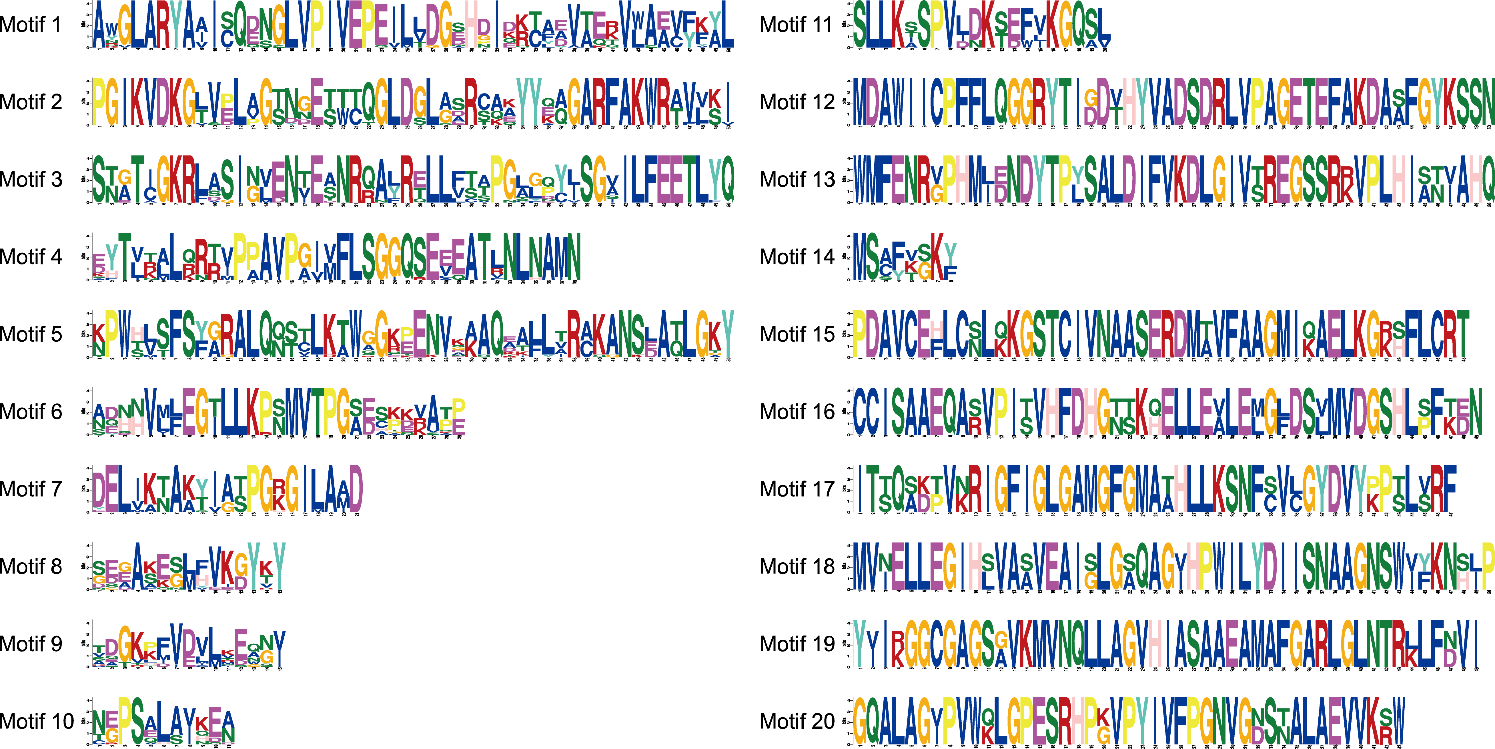


**
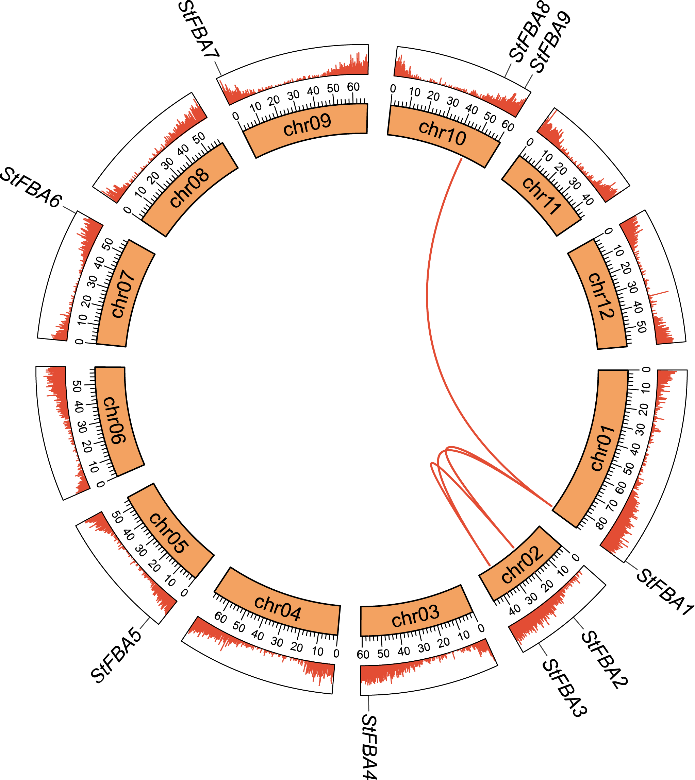
Supplementary Figure S3**. Intraspecific collinearity of the StFBA gene family members. The outer circle shows gene density information and inner circle indicates the number of chromosomes in the potato genome. The scale inside the circle indicates chromosome length. Duplicated gene pairs are linked with red line.

**Supplementary Figure S4**. Microtubers grown for 80 d under different spectrum. Scale bar 1 cm.


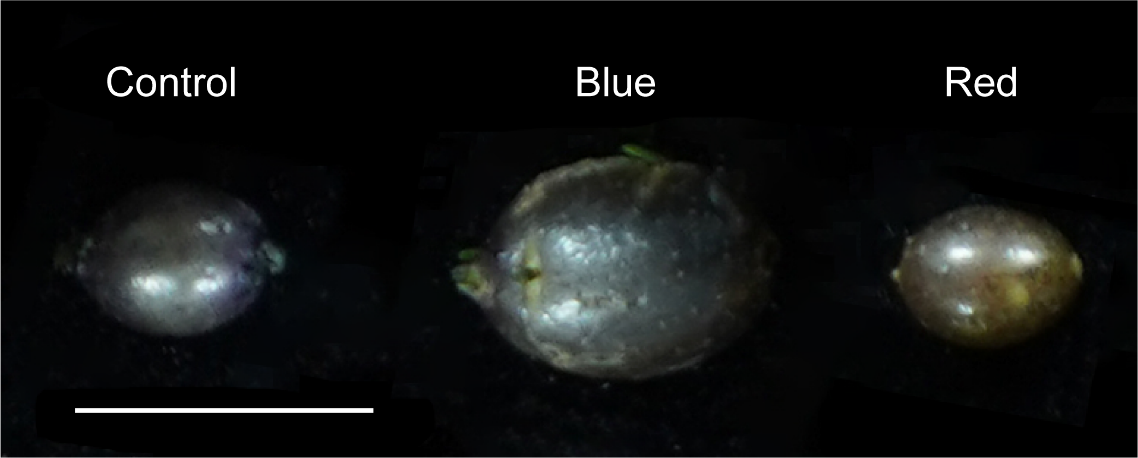

Supplement: Supplementary file 1 [file DataSheet1.ZIP › Supplementary Figures and Tables Captions.docx]
