# Supplementary material for: Characterization of FBA genes in potato (Solanum tuberosum L.) and expression patterns in response to light spectrum and abiotic stress
Source: Front Genet. 2024 Apr 12;15:1364944. doi: 10.3389/fgene.2024.1364944 (PMC11057440; doi:10.3389/fgene.2024.1364944)
Supplement: Supplementary file 1 [file DataSheet1.ZIP › Table S1.docx]

Table S1. FBA gene family profiles of different species

| Gene Name | | Gene ID | Amino acid number/aa | | Subcellular localization | |
| --- | --- | --- | --- | --- | --- | --- |
| *ALDY* | LOC Os06g40640.1 | | | 358 | | Cytoplasm |
| *OsFBA1* | LOC_Os01g67860.1 | | | 358 | | Cytoplasm |
| *OsFBA2* | LOC_Os05g33380.1 | | | 358 | | Cytoplasm |
| *OsFBA3* | LOC_Os10g08022.1 | | | 358 | | Cytoplasm |
| *OsFBA4* | LOC_Os08g02700.1 | | | 362 | | Cytoplasm |
| *OsFBA5* | LOC_Os01g02880.1 | | | 388 | | Chloroplast |
| *OsFBA6* | LOC_Os11g07020.1 | | | 388 | | Chloroplast |
| *TaFBA1* | LOC123059824 | | | 387 | | Chloroplast |
| *TaFBA2* | LOC542930 | | | 385 | | Chloroplast |
| *TaFBA3* | LOC123076846 | | | 387 | | Chloroplast |
| *TaFBA4* | LOC123086572 | | | 388 | | Chloroplast |
| *TaFBA5* | LOC123091418 | | | 388 | | Chloroplast |
| *TaFBA6* | LOC100415821 | | | 388 | | Chloroplast |
| *TaFBA7* | LOC123102769 | | | 385 | | Chloroplast |
| *TaFBA8* | LOC123111000 | | | 385 | | Chloroplast |
| *TaFBA9* | LOC123120002 | | | 385 | | Chloroplast |
| *TaFBA10* | LOC123062806 | | | 358 | | Cytoplasm |
| *TaFBA11* | LOC123062809 | | | 358 | | Cytoplasm |
| *TaFBA12* | LOC123071650 | | | 358 | | Cytoplasm |
| *TaFBA13* | LOC123071651 | | | 358 | | Cytoplasm |
| *TaFBA14* | LOC123079989 | | | 358 | | Cytoplasm |
| *TaFBA15* | LOC123079984 | | | 358 | | Cytoplasm |
| *TaFBA16* | LOC123079991 | | | 358 | | Cytoplasm |
| *TaFBA17* | gnl\|TA_TGACv1.30.dna.genome\|  TGACv1_scaffol d_394081_5AS  dna: scaffold: 1:45711:1 | | | 244 | | Cytoplasm |
| *TaFBA18* | TGACv1_scaffold_423736_5BS | | | 519 | | Cytoplasm |
| *TaFBA19* | LOC123149877 | | | 1383 | | Chloroplast |
| *TaFBA20* | LOC123160810 | | | 1383 | | Chloroplast |
| *TaFBA21* | LOC123169616 | | | 1383 | | Chloroplast |
| *AtFBA1* | AT2G21330 | | | 399 | | Chloroplast |
| *AtFBA2* | At4G38970 | | | 398 | | Chloroplast |
| *AtFBA3* | AT2G01140 | | | 391 | | Chloroplast |
| *AtFBA4* | AT5G03690 | | | 393 | | Mitochondrion |
| *AtFBA5* | AT4G26530 | | | 358 | | Cytoplasm |
| *AtFBA6* | AT2G36460 | | | 358 | | Cytoplasm |
| *AtFBA7* | AT4G26520 | | | 358 | | Cytoplasm |
| *AtFBA8* | AT3G52930 | | | 358 | | Cytoplasm |
| *SlFBA1* | Solyc01g110360.2.1 | | | 397 | | Chloroplast |
| *SlFBA2* | Solyc02g062340.2.1 | | | 395 | | Chloroplast |
| *SlFBA3* | Solyc02g084440.2.1 | | | 392 | | Chloroplast |
| *SlFBA4* | Solyc05g008600.2.1 | | | 395 | | Chloroplast |
| *SlFBA5* | Solyc10g054390.1.1 | | | 136 | | Chloroplast |
| *SlFBA6* | Solyc07g065900.2.1 | | | 431 | | Cytoplasm |
| *SlFBA7* | Solyc09g009260.2.1 | | | 358 | | Cytoplasm |
| *SlFBA8* | Solyc10g083570.1.1 | | | 358 | | Cytoplasm |
| *NtFBA1* | gene_57322 | | | 397 | | Chloroplast |
| *NtFBA2* | gene_38021 | | | 397 | | Chloroplast |
| *NtFBA3* | gene_60848 | | | 398 | | Chloroplast |
| *NtFBA4* | gene_73919 | | | 398 | | Chloroplast |
| *NtFBA5* | gene_62167 | | | 395 | | Chloroplast |
| *NtFBA6* | gene_35220 | | | 246 | | Chloroplast |
| *NtFBA7* | gene_68314 | | | 396 | | Chloroplast |
| *NtFBA8* | gene_3622 | | | 396 | | Chloroplast |
| *NtFBA9* | gene_54243 | | | 395 | | Chloroplast |
| *NtFBA10* | gene_20790 | | | 226 | | Chloroplast |
| *NtFBA11* | gene_38975 | | | 358 | | Cytoplasm |
| *NtFBA12* | gene_22224 | | | 358 | | Cytoplasm |
| *NtFBA13* | gene_67031 | | | 357 | | Cytoplasm |
| *NtFBA14* | gene_41189 | | | 357 | | Cytoplasm |
| *NtFBA15* | gene_76667 | | | 358 | | Cytoplasm |
| *NtFBA16* | gene_37698 | | | 358 | | Cytoplasm |
| *SmFBA1* | Smechr0100179.1 | | | 395 | | Chloroplast |
| *SmFBA2* | Smechr0200681.1 | | | 395 | | Chloroplast |
| *SmFBA3* | Smechr0202275.1 | | | 395 | | Chloroplast |
| *SmFBA4* | Smechr0702691.1 | | | 357 | | Mitochondrion |
| *SmFBA5* | Smechr0900105.1 | | | 358 | | Cytoplasm |
| *SmFBA6* | Smechr1000050.1 | | | 392 | | Chloroplast |
| *SmFBA7* | Smechr1002525.1 | | | 358 | | Mitochondrion |
