# Supplementary material for: Characterization of FBA genes in potato (Solanum tuberosum L.) and expression patterns in response to light spectrum and abiotic stress
Source: Front Genet. 2024 Apr 12;15:1364944. doi: 10.3389/fgene.2024.1364944 (PMC11057440; doi:10.3389/fgene.2024.1364944)
Supplement: Supplementary file 1 [file DataSheet1.ZIP › Table S2.docx]

Table S2 Prediction of secondary structure of potato FBA protein

| Name | Alpha helix | Beta turn | Random coil | Extended strand |
| --- | --- | --- | --- | --- |
| StFBA1 | 49.62% | 6.55% | 29.72% | 14.11% |
| StFBA2 | 45.82% | 6.08% | 33.16% | 14.94% |
| StFBA3 | 42.77% | 7.07% | 33.76% | 16.40% |
| StFBA4 | 46.48% | 8.27% | 29.01% | 16.24% |
| StFBA5 | 47.59% | 6.84% | 30.63% | 14.94% |
| StFBA6 | 53.22% | 7.28% | 27.17% | 12.32% |
| StFBA7 | 51.68% | 5.87% | 29.05% | 13.41% |
| StFBA8 | 49.42% | 5.26% | 30.41% | 14.91% |
| StFBA9 | 51.12% | 7.26% | 28.21% | 13.41% |
