# Supplementary material for: Characterization of FBA genes in potato (Solanum tuberosum L.) and expression patterns in response to light spectrum and abiotic stress
Source: Front Genet. 2024 Apr 12;15:1364944. doi: 10.3389/fgene.2024.1364944 (PMC11057440; doi:10.3389/fgene.2024.1364944)
Supplement: Supplementary file 1 [file DataSheet1.ZIP › Table S3.docx]

| Table S3. Gene duplication of *FBA* gene in *Solanum tuberosum* | | | | |
| --- | --- | --- | --- | --- |
| Gene Name | Gene ID | Gene Name | Gene ID | Duplication type |
| *StFBA1* | Soltu.DM.01G050210.1.v6.1 | *StFBA2* | Soltu.DM.02G006160.1.v6.1 | WGD or Segmental |
| *StFBA2* | Soltu.DM.02G006160.1.v6.1 | *StFBA3* | Soltu.DM.02G024280.2.v6.1 | WGD or Segmental |
| *StFBA3* | Soltu.DM.02G024280.2.v6.1 | *StFBA1* | Soltu.DM.01G050210.1.v6.1 | WGD or Segmental |
| *StFBA8* | Soltu.DM.10G016600.1.v6.1 | *StFBA1* | Soltu.DM.01G050210.1.v6.1 | WGD or Segmental |
