# Supplementary material for: Characterization of FBA genes in potato (Solanum tuberosum L.) and expression patterns in response to light spectrum and abiotic stress
Source: Front Genet. 2024 Apr 12;15:1364944. doi: 10.3389/fgene.2024.1364944 (PMC11057440; doi:10.3389/fgene.2024.1364944)
Supplement: Supplementary file 1 [file DataSheet1.ZIP › Table S4.docx]

Table S4. Information of primer sequence

| Gene | Primer sequence (5′→3′) |
| --- | --- |
| *StFBA1* | *GTCTACCGTTGATGGACGCA* |
|  | *TGGTAGTATGCAGCAGAGCG* |
| *StFBA2* | *TAAAAGGTCAGGCACTCCGC* |
|  | *GATGCAACAGTTTTCGCGGT* |
| *StFBA3* | *CTTTGAAGTCGCCAAGCAGG* |
|  | *GTCCACCAGACAAAAATTGCC* |
| *StFBA4* | *TGGCGCACTCGAAAAAGATG* |
|  | *ACACAAGCCATAGAGGTCCTTC* |
| *StFBA5* | *GCAACTGCCGGAAAGAGACTG* |
|  | *TAGGGGAACCAAACCCTTGTC* |
| *StFBA6* | *AAAGCAGGTGCTCGTTTTGC* |
|  | *GGCTCCACAATTGGCACAAG* |
| *StFBA7* | *GTACCCATTGTTGAGCCCGA* |
|  | *GAACAGCAGGTGGCATTGTG* |
| *StFBA8* | *GTGGCACATGTCCTTCTCCT* |
|  | *AAGGCCTTCTTCGGTGTACT* |
| *StFBA9* | *CGACAAGGGTACCGTAGAG* |
|  | *TCAGGCTCAACAATGGGGAC* |
| *EF-1-alpha* | *GATGGTCAGACCCGTGAACA* |
|  | *CCTTGGAGTACTTCGGGGTG* |

>S.tuberosum v6.1|Soltu.DM.01G050210|chr01:86885838..86892001

>S.tuberosum v6.1|Soltu.DM.02G006160|chr02:19797274..19799587

>S.tuberosum v6.1|Soltu.DM.02G024280|chr02:37587897..37590299

>S.tuberosum v6.1|Soltu.DM.03G033020|chr03:56749621..56770750

>S.tuberosum v6.1|Soltu.DM.05G004320|chr05:3719005..3722380

>S.tuberosum v6.1|Soltu.DM.07G027740|chr07:56783827..56789828

>S.tuberosum v6.1|Soltu.DM.09G004030|chr09:3432082..3434410

>S.tuberosum v6.1|Soltu.DM.10G016600|chr10:46399698..46402885

>S.tuberosum v6.1|Soltu.DM.10G024820|chr10:56467722..56471929
