# Supplementary figures and images for: Characterization of FBA genes in potato (Solanum tuberosum L.) and expression patterns in response to light spectrum and abiotic stress
Source: Front Genet. 2024 Apr 12;15:1364944. doi: 10.3389/fgene.2024.1364944 (PMC11057440; doi:10.3389/fgene.2024.1364944)

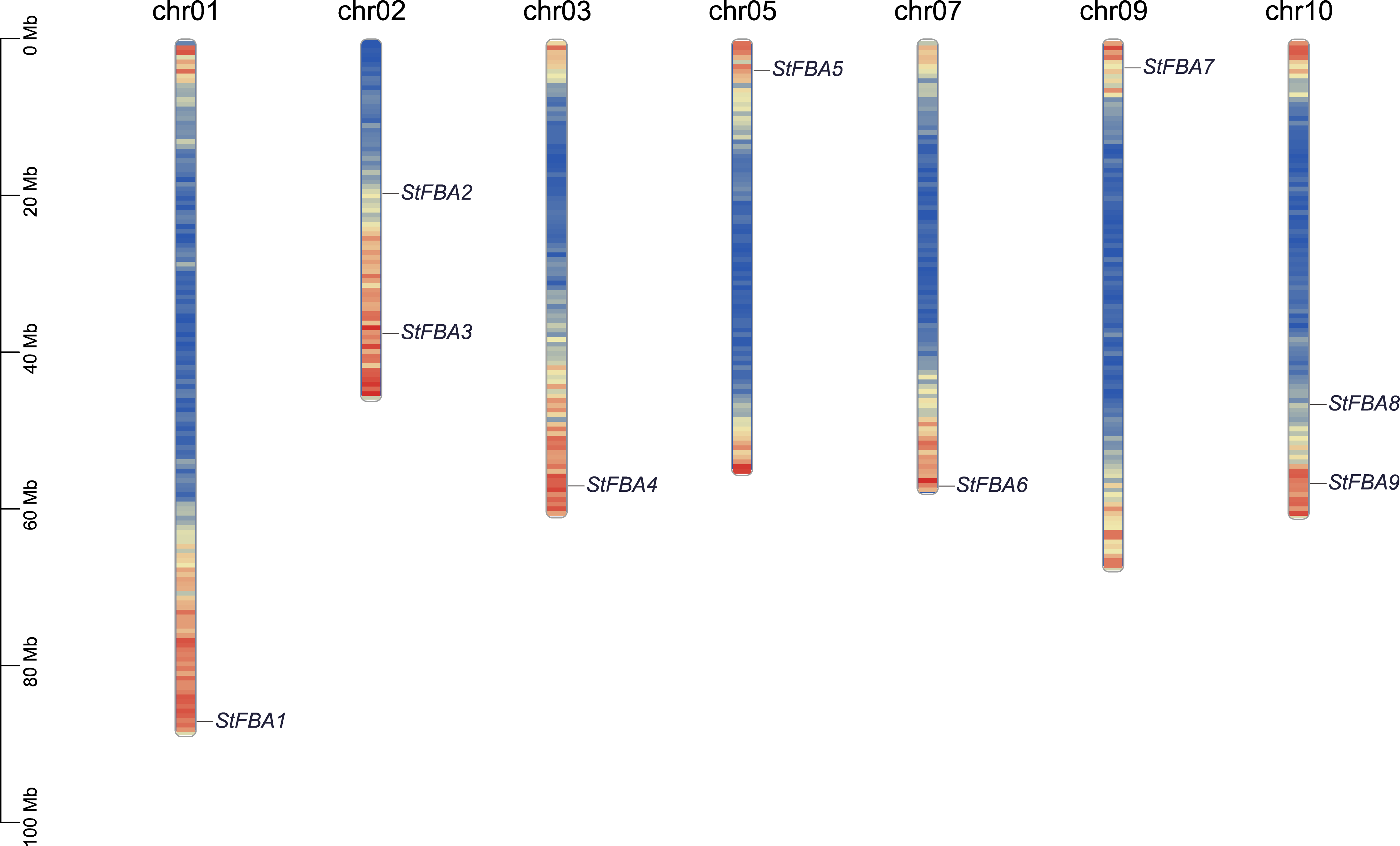

Supplement: Supplementary file 1 [file DataSheet1.ZIP › Figure S1.tif]

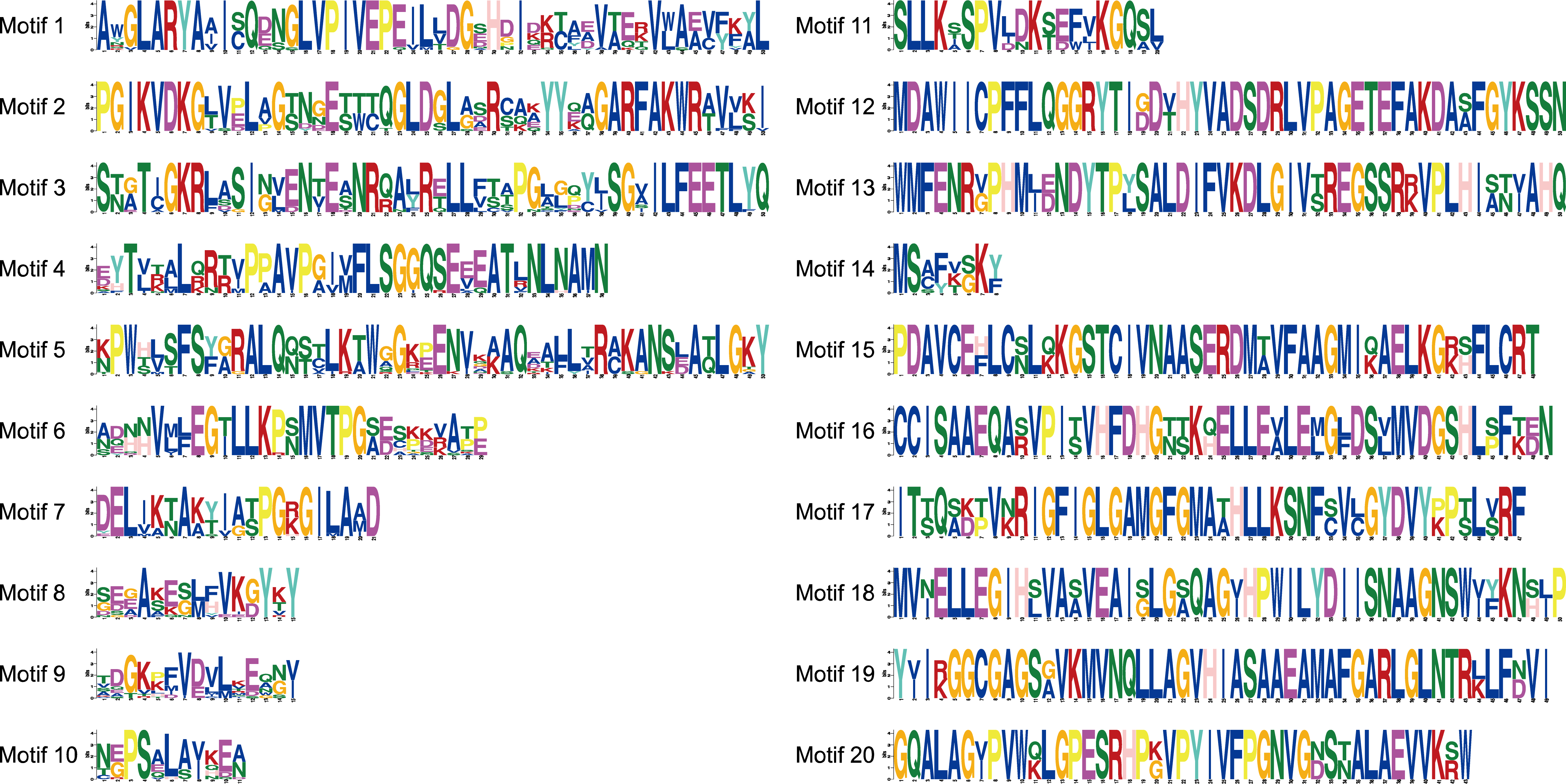

Supplement: Supplementary file 1 [file DataSheet1.ZIP › Figure S2.tif]

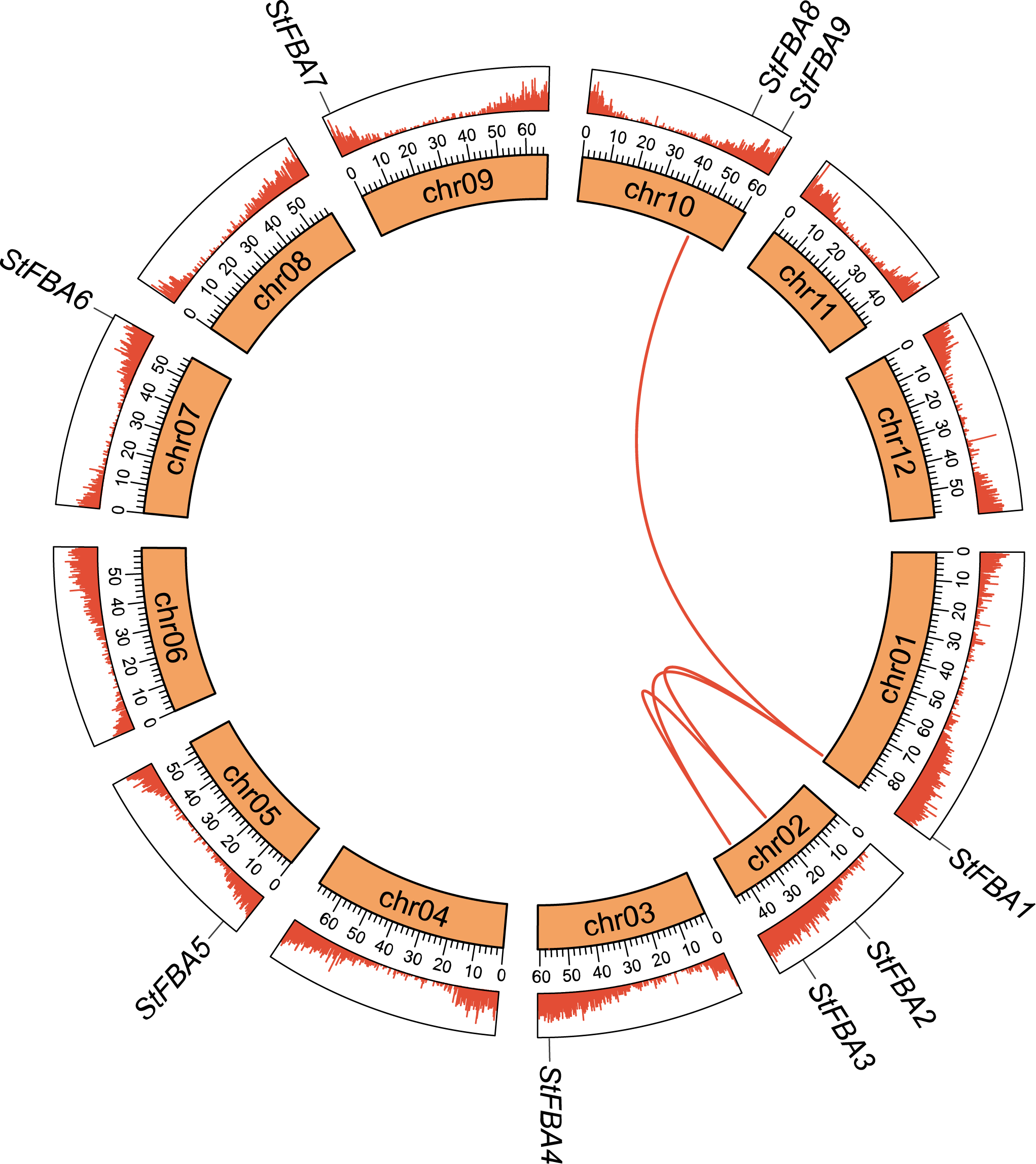

Supplement: Supplementary file 1 [file DataSheet1.ZIP › Figure S3.tif]

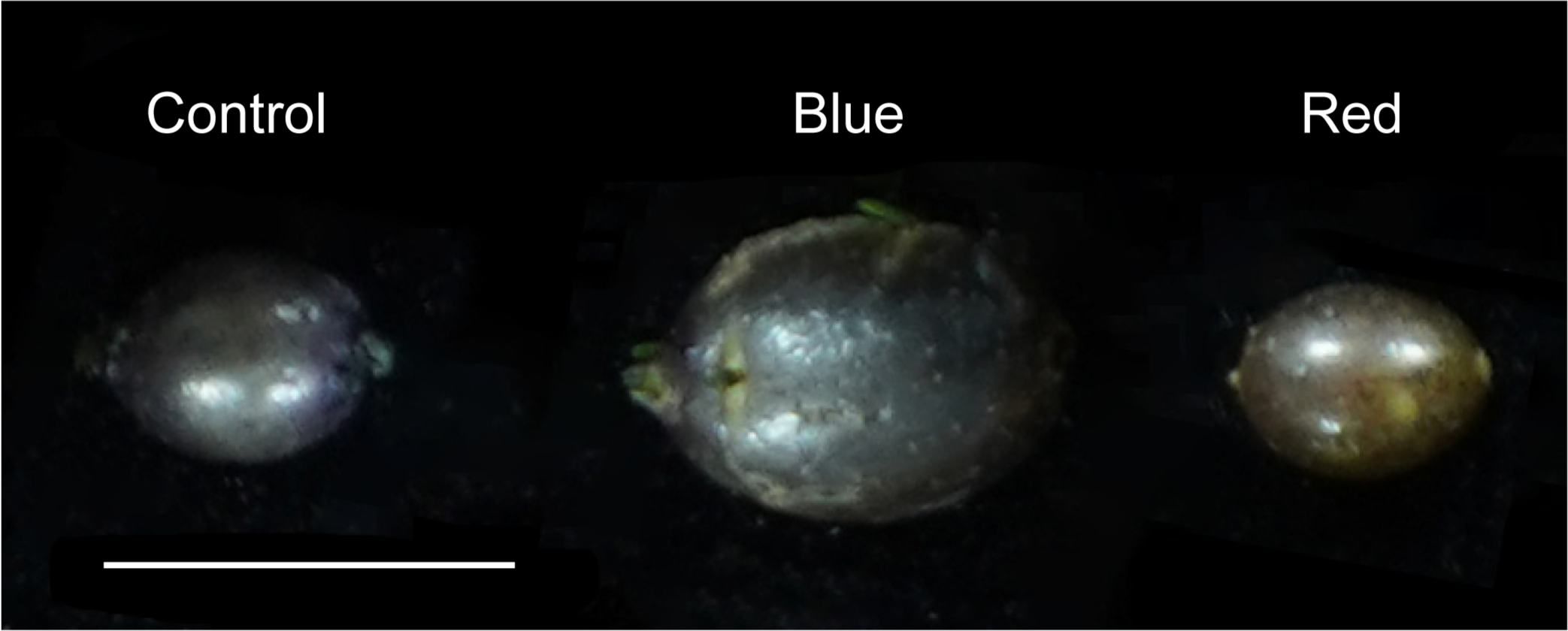

Supplement: Supplementary file 1 [file DataSheet1.ZIP › Figure S4.tif]
